# Human Biomechanical and Cardiopulmonary Responses to Partial Gravity – A Systematic Review

**DOI:** 10.3389/fphys.2017.00583

**Published:** 2017-08-15

**Authors:** Charlotte Richter, Bjoern Braunstein, Andrew Winnard, Mona Nasser, Tobias Weber

**Affiliations:** ^1^Space Medicine Office (HRE-AM), European Astronaut Centre Department (HRE-A) Cologne, Germany; ^2^Institute of Biomechanics und Orthopaedics, German Sport University Cologne, Germany; ^3^Centre for Health and Integrative Physiology in Space Cologne, Germany; ^4^German Research Centre for Elite Sport Cologne, Germany; ^5^Faculty of Health and Life Sciences, Northumbria University Newcastle upon Tyne, United Kingdom; ^6^Peninsula Dental School, Plymouth University Plymouth, United Kingdom; ^7^KBRwyle, Wyle Laboratories GmbH, Science, Technology and Engineering Group Cologne, Germany

**Keywords:** partial gravity, lunar gravity, martian gravity, biomechanics, energetics, exercise countermeasures

## Abstract

The European Space Agency has recently announced to progress from low Earth orbit missions on the International Space Station to other mission scenarios such as exploration of the Moon or Mars. Therefore, the Moon is considered to be the next likely target for European human space explorations. Compared to microgravity (μg), only very little is known about the physiological effects of exposure to partial gravity (μg < partial gravity <1 g). However, previous research studies and experiences made during the Apollo missions comprise a valuable source of information that should be taken into account when planning human space explorations to reduced gravity environments. This systematic review summarizes the different effects of partial gravity (0.1–0.4 g) on the human musculoskeletal, cardiovascular and respiratory systems using data collected during the Apollo missions as well as outcomes from terrestrial models of reduced gravity with either 1 g or microgravity as a control. The evidence-based findings seek to facilitate decision making concerning the best medical and exercise support to maintain astronauts' health during future missions in partial gravity. The initial search generated 1,323 publication hits. Out of these 1,323 publications, 43 studies were included into the present analysis and relevant data were extracted. None of the 43 included studies investigated long-term effects. Studies investigating the immediate effects of partial gravity exposure reveal that cardiopulmonary parameters such as heart rate, oxygen consumption, metabolic rate, and cost of transport are reduced compared to 1 g, whereas stroke volume seems to increase with decreasing gravity levels. Biomechanical studies reveal that ground reaction forces, mechanical work, stance phase duration, stride frequency, duty factor and preferred walk-to-run transition speed are reduced compared to 1 g. Partial gravity exposure below 0.4 g seems to be insufficient to maintain musculoskeletal and cardiopulmonary properties in the long-term. To compensate for the anticipated lack of mechanical and metabolic stimuli some form of exercise countermeasure appears to be necessary in order to maintain reasonable astronauts' health, and thus ensure both sufficient work performance and mission safety.

## Introduction

It is almost 50 years since July 1969, when Apollo 11 Astronauts Neil Armstrong and “Buzz” Aldrin were the first human beings to set foot on the Moon. The Apollo missions can still be regarded as one of the most exceptional endeavors in human history, not only from an engineering and technology perspective but also from a medical and physiological point of view. It was shown that the human body can adapt to extreme environments outside of Earth's protecting atmosphere and its gravitational field with an acceleration of 9.8 ms^−2^ (also referred to as 1 g). The Apollo Astronauts were able to live and work in micro- and partial gravity without experiencing any significant medical problems, neither during their (relatively short) missions nor upon their return to Earth (Berry, 1974).

In 2016 the Director General of the European Space Agency (ESA) introduced the agency's plans for the era after the planned decommissioning of the International Space Station (ISS) in 2024. The plans included going back to the Moon to set up a permanent habitat on its surface and/or a Cis-Lunar space station orbiting the Moon (Foing, 2016). It is thought that a progressively staggered approach using the proposed Lunar base will allow safer development and testing of hardware and procedures, toward the ultimate goal of a human space mission to Mars (Horneck et al., 2003; Goswami et al., 2012).

Astronauts exposed to microgravity (μg) experience physiological deconditioning (referred to as “space deconditioning”), in particular with regards to the physiological systems sensitive to mechanical loading such as the cardiovascular, pulmonary, neurovestibular, and musculoskeletal systems (Baker et al., 2008). In order to attenuate these effects, current ISS crew members exercise every day for 2.5 h including preparation time. Current exercise devices used on the ISS are a cycle ergometer and a treadmill for cardiovascular exercise (~1 h) as well as an advanced resistive exercise device (ARED) for strength training (~1.5 h) (Loehr et al., 2015; Petersen et al., 2016). Despite the extensive use of exercise countermeasures, astronauts still return from 6 months ISS missions showing space deconditioning effects. Examples of these effects include decreased calf muscle volume and power, loss of bone mineral density and reduction of peak oxygen uptake (Trappe et al., 2009; Moore et al., 2014; Sibonga et al., 2015).

It is understandable that in the past, medical divisions of space agencies have mainly set their foci of interest on the physiological effects of μg, to optimize operational procedures, to better understand the effects of μg on the human body and to mitigate undesirable and harmful effects. Consequently, compared to the bulk of literature and knowledge generated on the physiological effects of μg, the consequences of immediate and chronic partial gravity exposure (μg < partial gravity <1 g) as present on the Moon (0.16 g) or Mars (0.38 g), are somewhat understudied (Horneck et al., 2003; Goswami et al., 2012; Widjaja et al., 2015).

**Figure 1:**
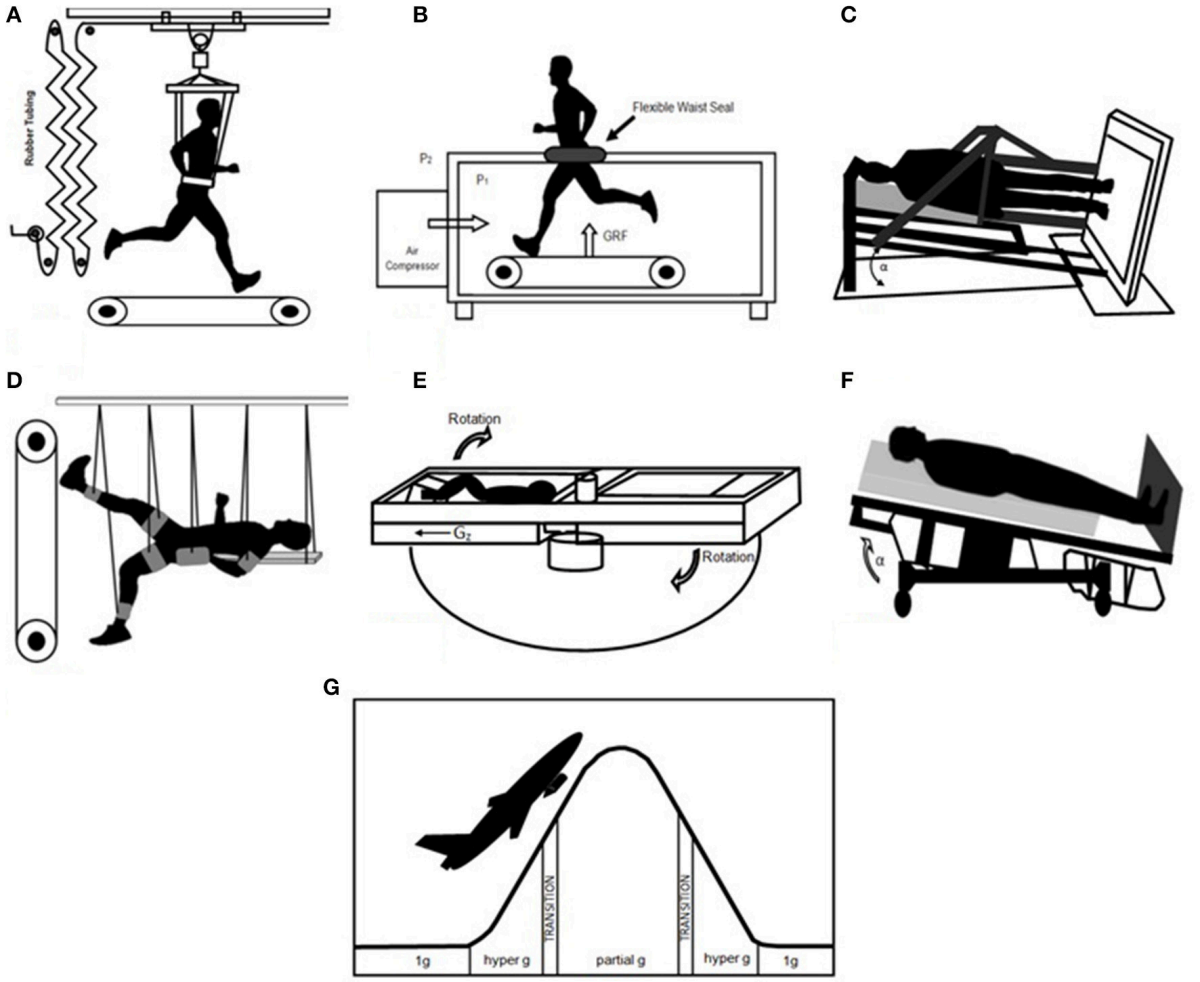
Partial gravity simulation models. **(A)** Vertical body weight support system (modified from Kram et al., 1997) **(B)** Lower body positive pressure treadmill (modified from Cutuk et al., 2006) **(C)** Tilted body weight support system (modified from Sylos-Labini et al., 2013) **(D)** Supine suspension system (modified from De Witt et al., 2008) **(E)** Centrifugation (modified from Katayama et al., 2004) **(F)** Head-up tilt (modified from Cavanagh et al., 2013) **(G)** Partial gravity parabolic flight (according to ESA 1st Joint European Partial Gravity Parabolic Flight Campaign, 2011).

Nonetheless, despite the fact that the knowledge gained through real partial gravity exposure during the Apollo missions and through partial gravity analogs is sparse, a first step to direct future research and to help to better understand physiological effects of partial gravity should be to gather and synthesize all available information of experiences made in the past. Logically, valuable sources of information are the medical data, records and publications of the Apollo missions conducted in the 1960s and 1970s with up to 75 h of continuous partial gravity exposure (Johnston and Hull, 1975; Kopanev and Yuganov, 1975) as well as various terrestrial partial gravity simulations (Figure 1; Shavelson, 1968; Davis and Cavanagh, 1993; Sylos-Labini et al., 2014; Salisbury et al., 2015).

The aim of this work was therefore to review all available information in order to quantify cardiopulmonary and biomechanical changes expected to occur in partial gravity environments (0.1–0.4 g). The objectives of the study were to:

1. Systematically review current evidence base to determine the human cardiopulmonary and biomechanical changes expected to occur in partial gravity.
2. Use effect sizes to enable direct comparisons of the differences between partial- and terrestrial gravity and pool results across multiple studies where possible.

Using the highest standard available to perform systematic reviews (www.cochrane.org) the synthesized information presented here shall help to identify knowledge gaps and develop a better understanding of medical issues that future astronauts will face when returning to the Moon and eventually advancing to Mars. Moreover, this systematic review seeks to provide a working reference for experts designing evidence-based exercise countermeasures for a Lunar habitat and future long-duration exploration missions beyond the Moon.

## Materials and methods

The present systematic review was conducted following the guidelines of the Cochrane Collaboration (Higgins and Green, 2011).

Additionally, the PRISMA (preferred reporting items for systematic reviews and meta-analyses) checklist was used to ensure transparent and complete reporting (Liberati et al., 2009).

### Search strategy

A range of keywords, grouped by main search terms, was used in various combinations to search the following databases for English language articles: Pubmed, Web of Science, Cochrane Collaboration Library, Institute of Electrical and Electronics Engineers database as well as ESA's “Erasmus Experiment Archive,” the National Aeronautics and Space Administration's (NASA) “Life Science Data Archive” and “Technical Reports Server” and the German Aerospace Centre's (DLR) database “elib”.

The literature search was performed in March and April 2016 according to the search strategy shown in Table 1. No restrictions to publication dates were applied. For ESA's, NASA's, and DLR's internal data archives, the search strategy was altered and specifically tailored due the inability to use “Boolean logic” in these databases. For the latter archives, only keywords of the search term “partial gravity” and/or one of the other synonyms (as listed in Table 1, search number 1) were used and all relevant records concerning biomechanics and/or the cardiopulmonary system were downloaded.

**Table 1:** Search strategy.

**Search number**	**Term**	**Keywords in Boolean search format**	**Search mask**
1	Partial gravity	“partial gravity” OR “fractional gravity” OR “reduced gravity” OR “lunar gravity” OR “moon gravity” OR “martian gravity” OR “mars gravity” OR “1/6th gravity” OR “1/6 G” OR “1/3rd gravity” OR “1/3 G” OR “low gravity” OR hypogravity OR “partial-gravity” OR “reduced-gravity” OR “Hypogravity” [Mesh:NoExp]	Title/ Abstract
2	Musculoskeletal	muscle^*^ OR muscle OR bone^*^ OR bone OR skeletal OR musculoskeletal OR “lean body mass” OR “body composition” OR osteo^*^ OR osteo OR “musculo-skeletal” OR neuromusculoskeletal OR “Musculoskeletal System” [Mesh]	All Fields
3	Cardiopulmonary	cardio^*^ OR cardio OR cardiac OR pulmona^*^ OR pulmonary OR cardiopulmonary OR cardiovascular OR vascular^*^ OR vascular OR respiratory OR respiration OR physiolog^*^ OR physiological OR physiology OR heart^*^ OR heart OR blood^*^ OR blood OR capillarisation OR capillary OR myocard^*^ OR myocard OR arterial OR venous OR orthostatic OR energetic^*^ OR energetic OR energy OR metabolic OR OR “Cardiovascular System” [Mesh] OR “Blood” [Mesh] OR “Circulatory and Respiratory Physiological Phenomena” [Mesh]	All Fields
4	Mechanics	biomechanic^*^ OR biomechanics OR mechanic^*^ OR mechanic OR locomotion OR gait OR walk^*^OR walk OR run^*^ OR run OR jump^*^ OR jump OR landing OR “ground reaction forces” OR impact^*^ OR impact OR “EMG” OR electromyo^*^ OR electromyography OR “mechanical work” OR kinetics OR kinematics OR workload OR power OR “Movement” [Mesh] OR “Mechanics” [Mesh] OR “Mechanical Phenomena” [Mesh]	All Fields
5	Partial g simulations and methods	(“body weight support” OR harness OR “alterG” OR “water immersion” OR “tilt table” OR “head-up tilt” OR “parabolic flight” OR “tail suspension” OR “supine suspension” OR “LBPP” OR “lower body positive pressure” OR “pressure suit” OR “subjects load device” OR centrifug^*^ OR centrifugation OR “vertical treadmill” OR exoskeleton) AND gravity	All Fields
7	Combined search	1 AND (2 OR 3 OR 4 OR 5)	

*Keywords were combined using the Boolean operators and grouped by main search terms. Medical Subject Headings (MeSH) as a comprehensive controlled vocabulary for the purpose of indexing journal articles and books in the life sciences were included in the search strategy. In the Pubmed advanced search builder either ‘Title/Abstract’ or ‘All Fields’ was used. The combined search allows to screen databases for various combinations of main search terms and their keywords*.

### Criteria for considering studies for this systematic review

The following eligibility criteria, which specify the types of included populations, interventions, control conditions, outcomes and study designs (PICOS) were applied.

#### Population

The main target group for the present systematic review were astronauts. However, since most of the included studies were simulation studies, healthy terrestrial people with no gender restrictions were included as well.

#### Interventions

Apollo missions 11–17 with Lunar surface time and various terrestrial partial gravity simulation models (Figure 1) were included (see list below). Variations in terms used for the different methods were at this point disregarded.

- Vertical body weight support systems
- Lower body positive pressure treadmills
- Tilted body weight support systems
- Supine suspension systems
- Centrifugation
- Head-up tilt
- Partial gravity parabolic flights

Only gravity levels from 0.1 up to 0.4 g were reviewed. Due to the varying gravity levels investigated in the reviewed studies, out of this range three different “gravity-groups” were determined with gravity conditions expressed either as the physical gravitational constant “g” or as percent of body weight (BW), applied body weight support (BWS) or degree of head-up tilt angles (HUT):

- Lunar gravity: 0.10–0.20 g 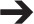 10–20% BW 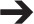 90–80% BWS 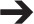 9.5–11° HUT
- Martian gravity: 0.30–0.40 g 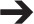 30–40% BW 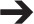 70–60% BWS 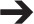 20–22° HUT
- In between: 0.25 g 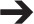 25% BW 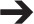 75% BWS 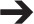 14.5° HUT.

#### Control conditions

Terrestrial gravity (1 g) and microgravity (μg) were used as control conditions.

#### Outcomes

To be included, studies had to contain outcomes linked to energetics and/ or biomechanics. A full list of outcome parameters is presented in Table 2.

**Table 2:** Outcome parameters for studies to be included.

**Field**	**Subfield**	**Specific outcome parameters**
Energetics	Cardiovascular and haemodynamic properties	heart rate, heart rate variability, stroke volume, cardiac output, ejection fraction, left ventricular systolic volumes, left ventricular diastolic volumes, left artrial dimension, systolic blood pressure, diastolic blood pressure, mean arterial blood pressure, pulse pressure, total peripheral resistance, blood flow volume, blood flow velocity, venous diameter, venous emptying volume, venous emptying time, bioelectrical impedance, blood volume, plasma volume, arteriovenous oxygen difference
	Pulmonary and metabolic properties	oxygen consumption, carbon dioxide production, respiratory rate, tidal volume, respiratory minute volume, respiratory quotient, respiratory gas exchange ratio, metabolic rate, locomotion efficiency, cost of transport
Biomechanics	Morphology	muscle volume, fiber type composition, physiological cross sectional area, anatomical cross sectional area, pennation angle, muscle fiber length, tendon stiffness, bone mineral density
	Kinematics	center of mass (CoM) velocity, CoM waveform, CoM energy level, CoM mechanical work, CoM mechanical power, joint angle, angular velocity, angular waveform, ground contact time, flight time, duty factor, frequency, cadence, stride length, step length, preferred walk-to-run transition speed
	Kinetics	muscle force, joint moments, joint stiffness, peaks and magnitudes of horizontal (mediolateral and anterior-posterior) ground reaction forces (GRF), vertical GRF, GRF impulses
	Activation Pattern	muscle activation pattern, H-Reflex and M-Wave (electrical peripheral nerve stimulation)

*Outcomes are divided into the two main groups “energetics” and “biomechanics” and further into subgroups with more specific outcome measurements*.

#### Study designs

All types of experimental studies were included.

### Data collection and analysis

#### Study selection

**Figure 2:**
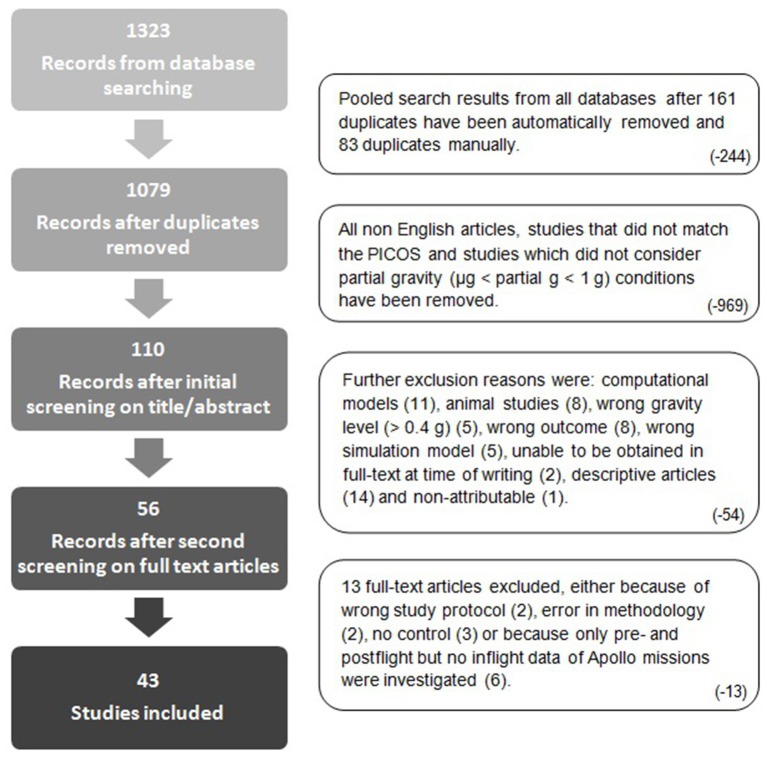
Search and screening strategy and numbers.

Studies were screened by the lead author and one other independent reviewer using the Rayyan web application (https://rayyan.qcri.org/) (Elmagarmid et al., 2014). The initial screening was performed using titles and abstracts. Considering the main research question of the present study (which human biomechanical and cardiopulmonary changes occur due to partial gravity exposure?) relevant articles were included. Articles were excluded if titles and/or abstracts were considered as clearly irrelevant. This was the case if titles and abstract did not reveal a direct link to the previously defined PICOS. Any uncertainties of study inclusion or exclusion were discussed consulting a third expert reviewer. Full-text articles were obtained in case the initial screening was unclear and were downloaded for all other included studies. After screening the full-text resources a further round of exclusion took place. The complete systematic literature screening and exclusion process is illustrated in Figure 2.

#### Data extraction and management

Data extraction from each study was performed using an adapted version of the Cochrane Collaboration's ‘Data collection form for intervention reviews: RCTs and non-RCTs’, version 3, April 2014 (RCT: randomized controlled trial).

#### Assessment of risk of bias in included studies

The Cochrane Collaboration's risk of bias analysis tool was used to assess the quality of included studies. Uncertainties were discussed with a third reviewer. As the study types of included studies were mainly case series without a separated control group [for study classification see also: “2009 Updated Method Guidelines for Systematic Reviews in the Cochrane Back Review Group” by Furlan et al. (2009)] and with small sample sizes, often not much information to allow for objective judgment was provided. A “+” stands for low risk, “−” for high risk and “?” for unclear risk. For the studies that are case series, no risks of random sequence generation and allocation concealment can be assessed (NA–not applicable, see Table 3).

**Table 3:** Summary of risk of bias.

	**Random sequence generation (selection bias)**	**Allocation concealment (selection bias)**	**Blinding of participants and personnel (performance bias)**	**Blinding of outcome assessment (detection bias)**	**Incomplete outcome data (attrition bias)**	**Selective outcome reporting (reporting bias)**
Aerts et al., 2012	NA	NA	**?**	**?**	**+**	**+**
Baranov et al., 2016	**?**	NA	**?**	**?**	**+**	**−**
Berry, 1969	NA	NA	NA	NA	**?**	**?**
Berry, 1970	NA	NA	NA	NA	**?**	**?**
Berry, 1974	NA	NA	NA	NA	**?**	**?**
Cardus, 1996	NA	NA	**?**	**?**	**+**	**−**
Cavagna et al., 2000	NA	NA	**?**	**?**	**+**	**+**
Cavanagh et al., 2013	NA	NA	**?**	**?**	**−**	**+**
Chang et al., 1996	NA	NA	**?**	**?**	**+**	**+**
Chang et al., 2001	NA	NA	**?**	**?**	**+**	**+**
Cowley et al., 2015	NA	NA	**?**	**?**	**+**	**+**
Cutuk et al., 2006	NA	NA	**?**	**?**	**+**	**+**
De Witt et al., 2014	NA	NA	**?**	**?**	**+**	**+**
Donelan and Kram, 1997	NA	NA	**?**	**?**	**+**	**+**
Donelan and Kram, 2000	NA	NA	**?**	**?**	**+**	**+**
Evans et al., 2013	NA	NA	**?**	**?**	**−**	**−**
Farley and McMahon, 1992	NA	NA	**?**	**?**	**+**	**+**
Ferris et al., 2001	NA	NA	**?**	**?**	**+**	**+**
Fox et al., 1975	NA	NA	**?**	**?**	**−**	**+**
Grabowski et al., 2005	NA	NA	**?**	**?**	**+**	**+**
Griffin et al., 1999	NA	NA	**?**	**?**	**+**	**−**
He et al., 1991	NA	NA	**?**	**?**	**+**	**+**
Ivanenko et al., 2002	NA	NA	**?**	**?**	**+**	**+**
Ivanenko et al., 2011	NA	NA	**?**	**?**	**+**	**+**
Kopanev and Yuganov, 1975	NA	NA	NA	NA	**?**	**?**
Kostas et al., 2014	NA	NA	**?**	**?**	**+**	**+**
Kram et al., 1997	NA	NA	**?**	**?**	**+**	**+**
Lathers et al., 1990, 1993; Lathers and Charles, 1994	NA	NA	**?**	**?**	**+**	**+**
Louisy et al., 1994	NA	NA	**?**	**?**	**+**	**+**
Pavei and Minetti, 2015	NA	NA	**?**	**?**	**+**	**+**
Pavei et al., 2015	NA	NA	**?**	**?**	**+**	**+**
Pavy-Le Traon et al., 1997	NA	NA	**?**	**?**	**+**	**+**
Robertson and Wortz, 1968	NA	NA	**?**	**?**	**+**	**+**
Schlabs et al., 2013	NA	NA	**?**	**?**	**−**	**+**
Spady and Harris, 1968	NA	NA	**?**	**?**	**+**	**+**
Spady and Krasnow, 1966	NA	NA	**?**	**?**	**+**	**+**
Sylos Labini et al., 2011	NA	NA	**?**	**?**	**+**	**+**
Sylos-Labini et al., 2013	NA	NA	**?**	**?**	**+**	**+**
Teunissen et al., 2007	NA	NA	**?**	**?**	**+**	**+**
Waligora and Horrigan, 1975	NA	NA	NA	NA	**?**	**?**
Widjaja et al., 2015	NA	NA	**?**	**?**	**−**	**−**

*Authors' judgement about each methodological quality item of each included study*.

*+: Low risk; −: High risk; ?: Unclear risk; NA: Not applicable*.

#### Quality appraisal of technical principles to simulate partial gravity

There is limited high quality research on changes in energetics and biomechanics in humans due to exposure to partial gravity. Main problems are logistical limitations, limited numbers of participants and a diversity of simulation models. Therefore, no tools for assessing partial gravity methodological quality are available except for the approach of Chappell and Klaus (2013) who characterized models allowing locomotion of being good or poor in reproducing factors associated with partial gravity (Chappell and Klaus, 2013). Since there was a lack of completeness in Chappell and Klaus (2013) it was decided for this review to develop a new rating scale of included technical principles with partial gravity parabolic flights set as a gold standard (see Table 4). The underlying assumption of this tool is how well the simulation study reflects the reality. This can provide an indicative rating how well the simulation study results are transferable to real human partial gravity missions. This tool is piloted in the present review to highlight which studies may have a greater rigor in simulating partial gravity but it is important to consider that no further empirical studies on its validity and reliability were performed.

**Table 4:** Quality appraisal of included technical principles to simulate the effects of partial gravity on the various physiological and biomechanical outcome measures.

**Methods/parameter**	**Cardiovascular properties**	**Respiratory & metabolic properties**	**Musculoskeletal properties**	**Kinematics**	**Ground reaction forces**	**Muscle activation pattern**	**Points**	**Ranking**
Partial gravity parabolic flight	x x x	x x x	x x x	x x x	x x x	x x x	18	1
Vertical body weight support systems	x	x x	x x	x x	x x x	x x	12	4
Lower body positive pressure treadmills	x x	x x	x x	x x	x x x	x x x	14	2
Tilted body weight support systems	x x	x x	x x	x x	x x x	x x	13	3
Supine suspension systems	x x	x x	x x	x x	x x x	x x	13	3
Head-up tilt	x x	x x	x x	x x	x x x	x x	13	3

*All methods were rated as per how accurate they might mimic the effects of partial gravity for relevant physiological and biomechanical categories. With: x: meaning not accurate; xx: quite accurate and xxx: very accurate. The sum of all x's results in the number of total points per method and therefore defines the overall ranking. Partial gravity parabolic flight was set as a gold standard*.

#### Data analysis

Main changes across all outcome measures are presented in six different tables (**Supplementary Tables**). There are three tables for cardiopulmonary changes and three tables for biomechanical changes presenting outcomes of the three defined gravity ranges (Lunar, in between and Martian -gravity). Changes from either terrestrial gravity and/or microgravity as control conditions are presented with arrows. An up (↑) or down (↓) arrow was set as soon as minimal changes of the mean were presented either as values or as figures (visual observation) or if the authors stated that values were in- or decreasing (even if not statistically significant or if no statistics were performed), meaning that there is an upward or downward trend. Arrows marked with an asterisk (^*^) indicate that there were statistically significant differences from control with *P* < 0.05. Arrows marked with a hash tag (#) indicate that only partly statistical significant differences were found, for example if in general values increased but only for men significantly. A horizontal arrow (→) was used if no visual differences were detected, values were the same or if the authors stated that there were no changes. A swung dash (~) was used in case of inconsistent results for example if two participants showed results in opposite direction.

Effect sizes (for data available either presented in included articles or obtained from authors after requested) were calculated between partial gravity conditions and 1 g. The effect sizes were then bias corrected using weighted (accounting for *n* = sample size) pooled standard deviations as per Hedge's g method (Ellis, 2010). Effect sizes in **Figures 5**–**9** are presented as Hedge's g:

Hedge's g= sample mean 2 -sample mean 1pooled standard deviation of sample 1 and 2(weighted)

In the absence of previously reported and validated minimal clinically meaningful changes on which to base conclusions, standardized mean changes between comparisons groups were defined. As there are currently no direct empirical studies for astronauts to demonstrate the thresholds suggested by Hopkins et al. (2009) were used. Thresholds of 0.1, 0.3, 0.5, 0.7, and 0.9 were defined as small, moderate, large, very large and extremely large effects between two comparison groups (Hopkins et al., 2009). This enabled conclusions to be based upon the estimated size of the effect between g-levels. The level for the confidence interval for the effect size comparisons was set to 95%. The most meaningful effect sizes are presented in plots to highlight the areas where medical operations will need to focus attention ahead of the missions taking place.

## Results

### Description of studies

The study selection process and reasons for exclusion are summarized in Figure 2. The initial search identified 1,323 citations of which 244 were confirmed to be duplicates. Therefore, 1,079 titles and abstracts were screened and further 969 studies excluded which did not meet the eligibility criteria. After reading the remaining 110 full-text articles, further 54 studies were excluded for various reasons (see description on the right side of the flow chart in Figure 2). Initially, 56 studies met the inclusion criteria but 13 of them were excluded after being defined as not suitable considering the protocol, methodology, control condition, or time points of data acquisition.

**Figure 3:**
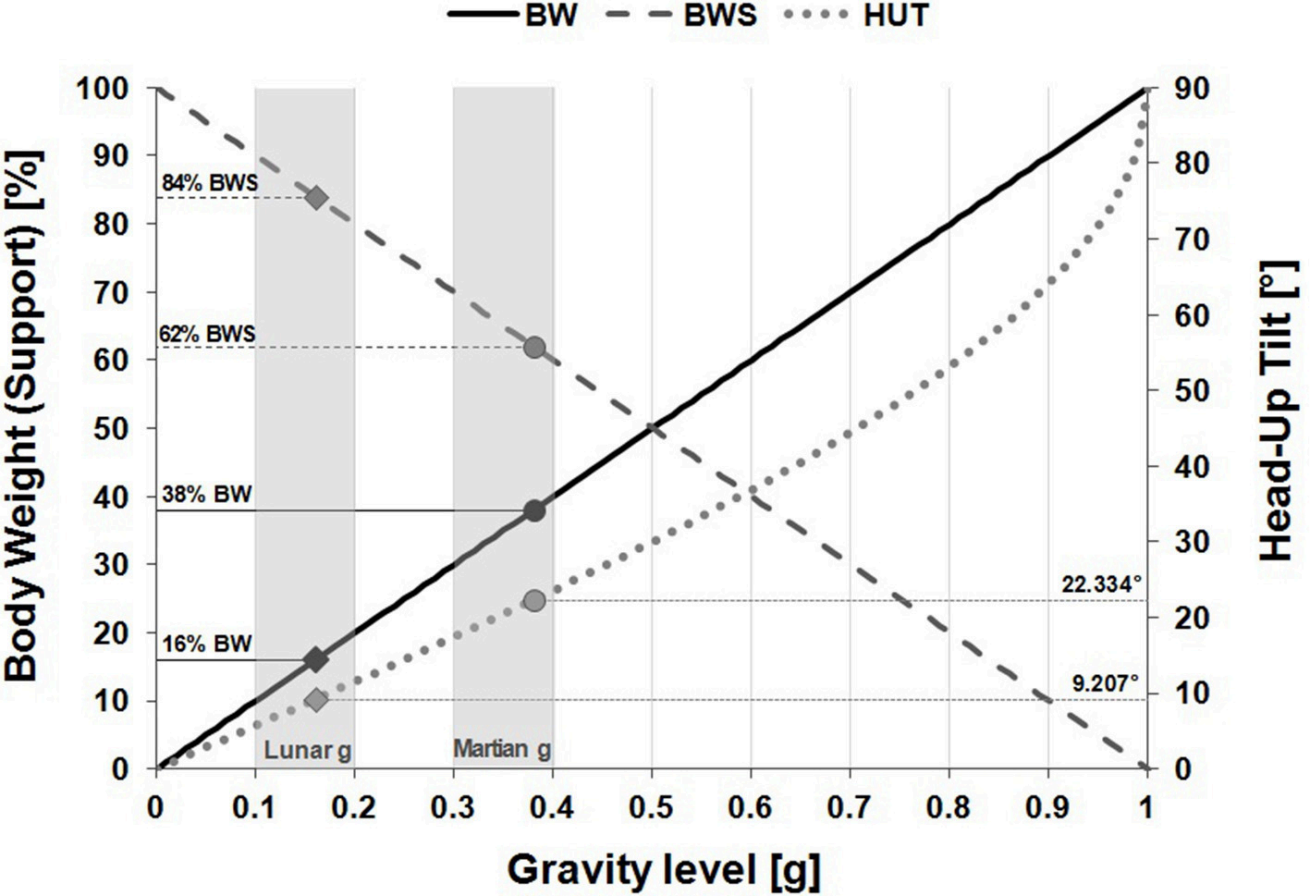
Various levels of gravity expressed in different units. The exact value for Lunar gravity is 0.16 g or 9.21° HUT or 16% BW or 84% BWS. The exact value for Martian gravity is 0.38 g or 22.33° HUT or 38% BW or 62% BWS. The ranges that were considered acceptable for Lunar- and Martian gravity in the present study are shown in gray. The exact values for Lunar and Martian gravity and each unit are depicted through solid diamonds and circles. BW, Body weight (in %); BWS, Body weight support (in %); HUT, Head-up tilt (in degree).

The final 43 included studies were mainly case series studies except for the case report of Waligora and Horrigan (1975) and the study of Baranov et al. (2016) who conducted a randomized controlled trial. Apart from the two latter publications, all other included studies investigated different levels of partial gravity without a separated control group. Depending on the technical principles used to simulate partial gravity and a different terminology, the authors expressed partial gravity either as percent of body weight, percent of body weight support, degree of head-up tilt or as a specific gravity level (g). For a uniform designation, Figure 3 helps to translate different units into the gravitational acceleration “g”, as it will be the standard unit used within this review.

**Figure 4:**
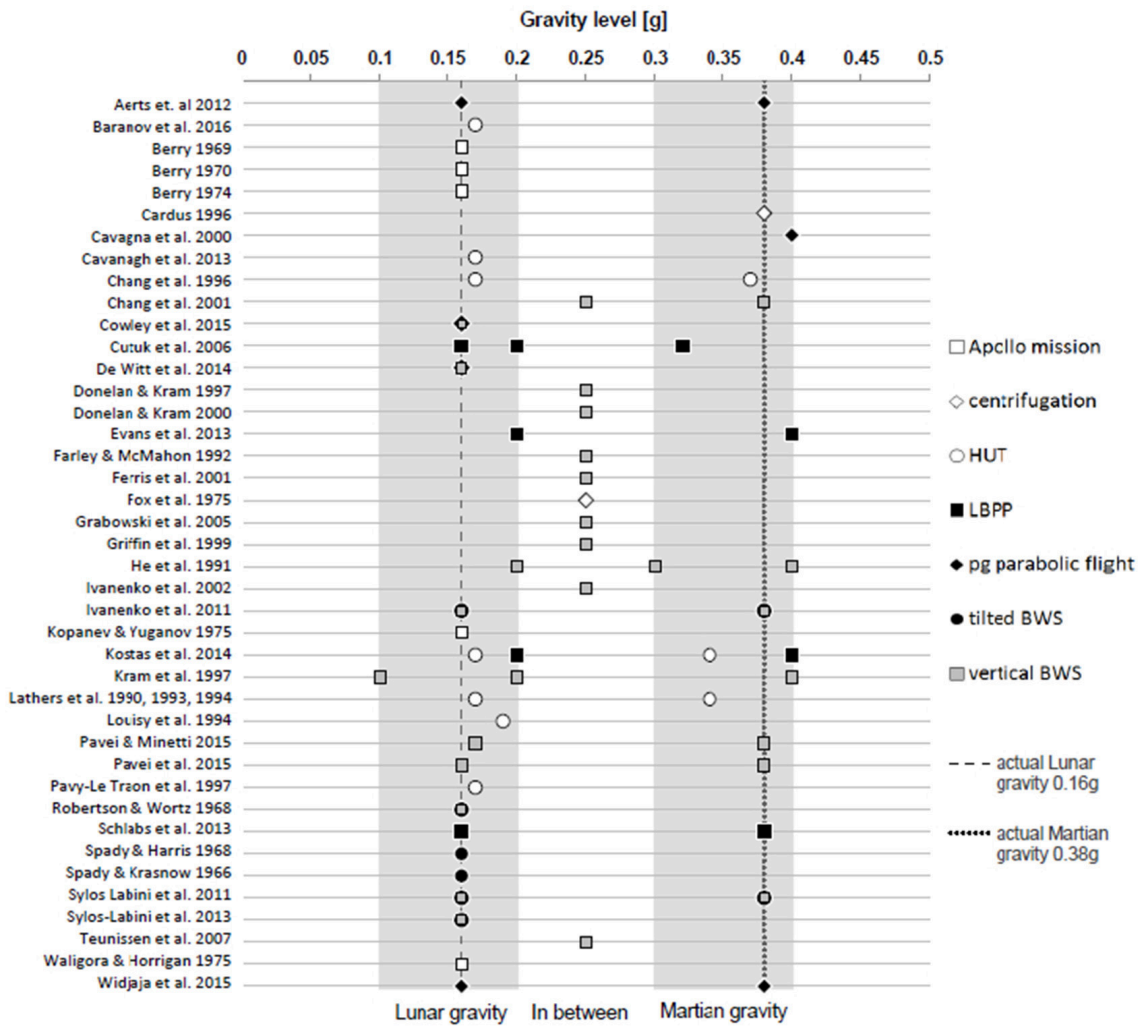
Gravity levels and simulation models of included studies. The ranges that were considered acceptable for Lunar- and Martian gravity in the present review are shown in gray. The exact values for Lunar and Martian gravity are depicted through dashed and dotted lines. Control conditions and measured gravity levels outside the defined range of 0.1–0.4 g are not shown. HUT, Head-up tilt; LBPP, Lower body positive pressure; BWS, body weight support system; pg, partial gravity.

Figure 4 summarizes the applied gravity levels within the in the PICOS defined gravity range (0.1–0.4 g) as well as the simulation model used of each included study. As shown in Figure 4, the majority of studies (*n* = 29) were conducted in the range of Lunar gravity (0.1–0.2 g). Out of these 29 studies, 18 applied actual Lunar gravity of 0.16 g. Seventeen studies were conducted in the range of Martian gravity and nine applied the actual value for Martian gravity. In the range between Lunar and Martian gravity 10 studies applied 0.25 g. Nine studies used μg as a comparison whereas 41 studies compared their outcomes to 1 g (under consideration that studies could use μg and/or 1 g as control conditions).

Nineteen studies used vertical body weight support systems (■), indicating that this was the most often used simulation model to generate partial gravity conditions. Additionally, in descending order, nine studies used head-up tilt (○), six studies tilted body weight support systems (•), five studies partial gravity parabolic flights (♦), four studies lower body positive pressure (■) and two studies centrifugation (♢). Physiological data of Lunar surface explorations during Apollo missions (□) were presented in five studies.

The age of participants across the studies ranged from 18–63 years. Studies recruited predominantly men. Taken together 197 men and 88 women participated in total. For 19 adults gender was not indicated. The highest number of participants within one study was 21, the lowest number was two.

Some of the investigated partial gravity simulation models did not allow movements. When movements were not possible (e.g., head-up tilt) or required, posture within the experimental protocols was semi-supine (a body position where the participant lies on his/her back but is not completely horizontally) sitting or standing. Included locomotion types were walking (^w^), running (^r^), skipping (^s^), and hopping (^h^) at different velocities or the preferred walk-to-run transition speed (^PTS^).

### Methodological quality of included studies

The overall risk of bias (see Table 3) was very low. Most studies were case series and did not have control groups, therefore, some aspects of the Cochrane risk of bias tool (which was designed for controlled clinical trials) were not relevant. This includes randomization and allocation concealment. In addition to this, many studies failed to give sufficient detail to assess their potential risk of bias including blinding of participants, personnel and outcome assessment. Therefore, only conclusions about incomplete outcome data and selective reporting could be drawn.

The majority of included studies were case series. Hence, the evidence/taxonomy of study designs of included studies is very low (IV, where V is the lowest level of evidence) using “Oxford Centre for Evidence-based Medicine” (March, 2009) guidelines (Phillips et al., 1998).

The number of participants in the included studies was comparably low and therefore often no adequate statistical analysis in consistency with the research question was performed. This reduces the quality of most of the included studies with respect to the authors' research question.

### Results of changes in outcome parameters

All characteristics of included studies and changes from 1 g and/or μg of relevant outcome parameters due to exposure to partial gravity are presented in Supplementary Tables 1–6.

**Figure 5:**
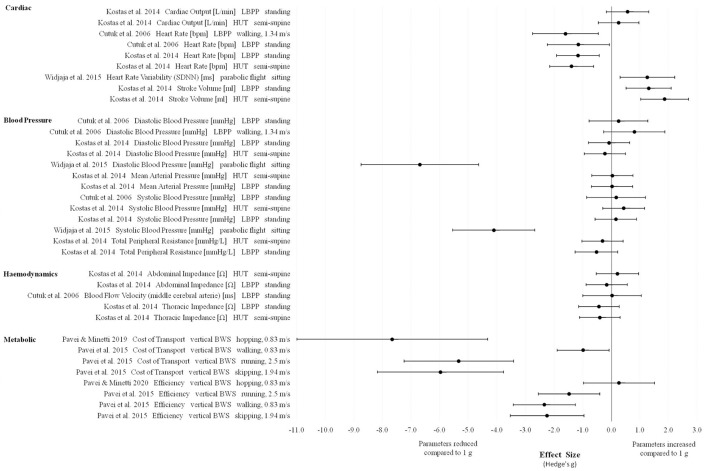
Effect sizes (Hedge's g) and confidence intervals for cardiopulmonary parameters in Lunar gravity compared to 1 g.

**Figure 6:**
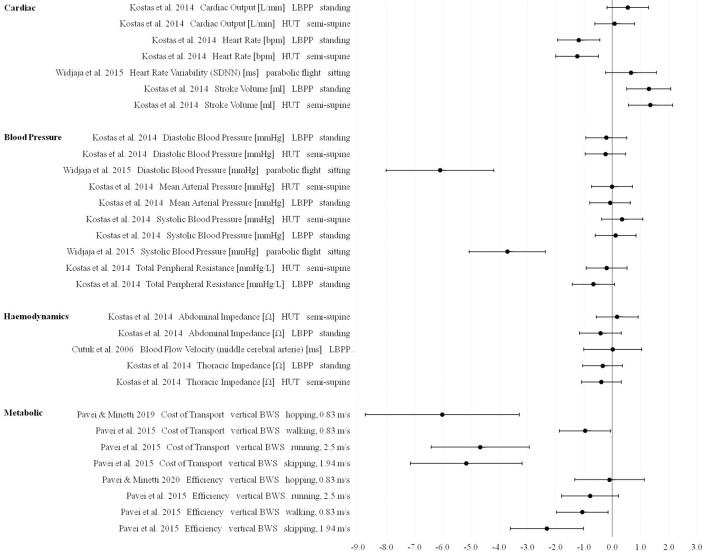
Effect sizes (Hedge's g) and confidence intervals for cardiopulmonary parameters in Martian gravity compared to 1 g.

**Figure 7:**
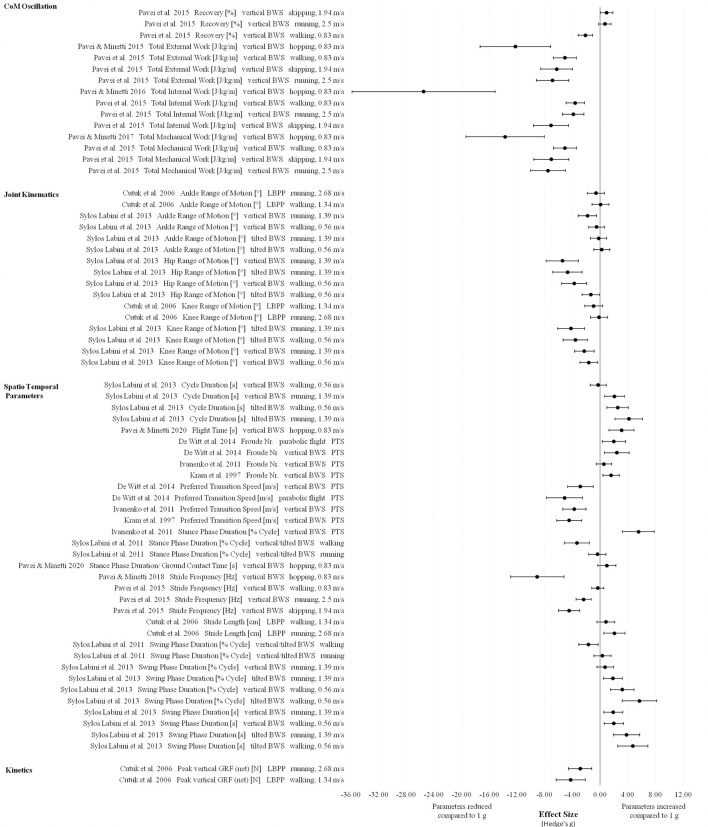
Effect sizes (Hedge's g) and confidence intervals for biomechanical parameters in Lunar gravity compared to 1 g.

**Figure 8:**
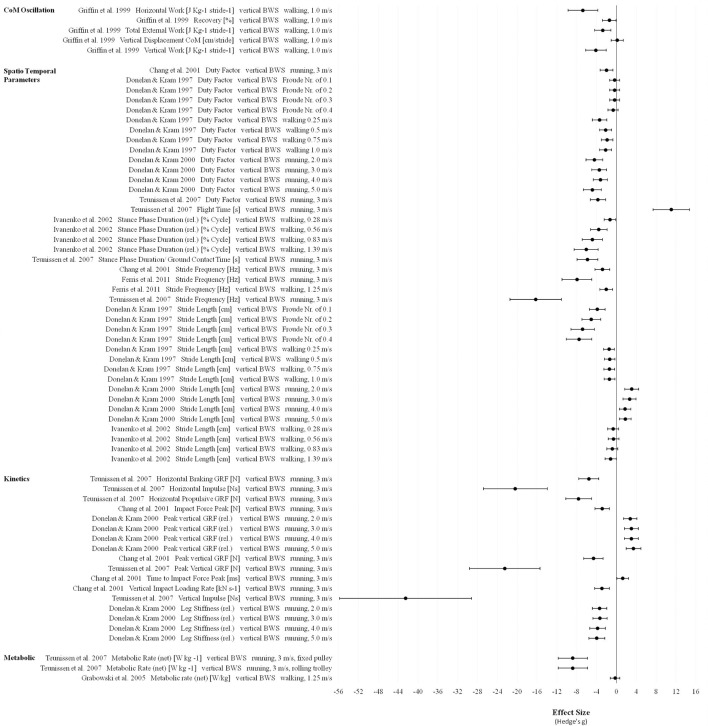
Effect sizes (Hedge's g) and confidence intervals for biomechanical and cardiopulmonary parameters in 0.25 g compared to 1 g.

**Figure 9:**
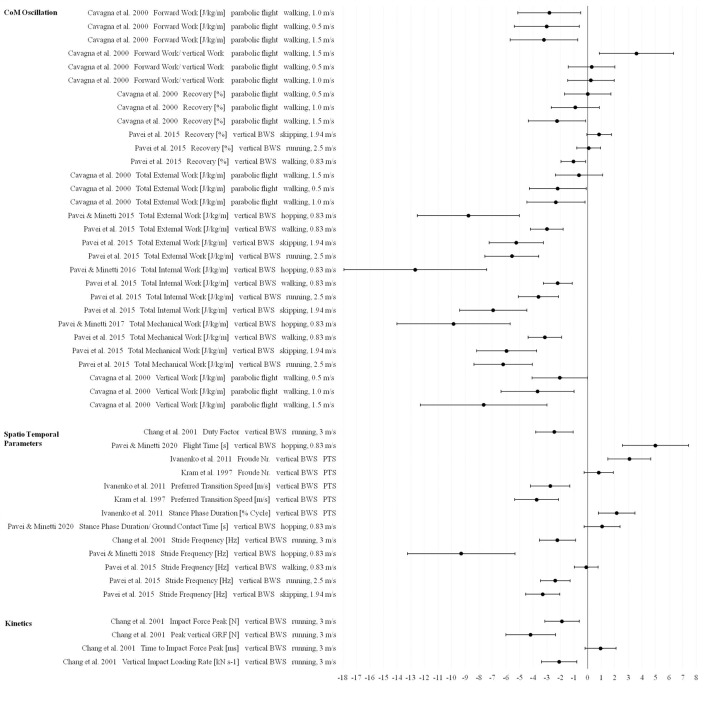
Effect sizes (Hedge's g) and confidence intervals for biomechanical parameters in Martian gravity compared to 1 g.

In the following the clinical relevance of available data is presented. Main effects of outcome parameters and their bias corrected effect sizes (Hedge's g) are depicted in Figures 5–**9**. Please note that since the scale we used to define effect sizes (as per Hopkins et al. (2009): small, moderate, large, very large and extremely large) we mainly found extremely large effect sizes, referring to effect sizes larger than 0.9. This means that differences of effect sizes within the category “extremely large” can be really great (as presented in Figures 5–**9**).

#### Main effects and effect sizes of cardiopulmonary changes in partial gravity

In the following, if effects were similar in direction and magnitude, then these effects were generalized and body postures and simulation models were not further considered.

Heart rate, stroke volume, cost of transport, efficiency (except of the hopping condition in Pavei and Minetti, 2015) as measured in Lunar and Martian gravity conditions revealed the most pronounced changes compared to 1 g (Figures 5, 6).

For cardiac output as measured using the lower body positive pressure model, Kostas et al. (2014) presented large effects in Lunar and Martian gravity. The effects for cardiac output as measured during head-up tilt revealed moderate changes in Lunar and small changes in Martian gravity compared to 1 g.

For blood pressure parameters, inconsistent results between the different included studies were found. Widjaja et al. (2015) presented extremely large effects for diastolic and systolic blood pressure values measured during a Lunar and Martian gravity parabolic flight. The study of Kostas et al. (2014) presented mostly small effects for different blood pressure parameters using two different simulation models. Using the lower body positive pressure model, effects for total peripheral resistance were reported to be moderate (Lunar vs. 1 g) and large (Martian vs. 1 g). For thoracic impedance, data published by Kostas et al. (2014) revealed moderate changes in Lunar and Martian gravities compared to 1 g.

For 0.25 g only effect sizes of net metabolic rates are presented (**Figure 8**). Teunissen et al. (2007) found extremely large effects during running while data from Grabowski et al. (2005) reported a small effect during walking in 0.25 g compared to 1 g.

#### Main effects and effect sizes of biomechanical changes in partial gravity

In all of the three defined gravity ranges (Figures 7–9) vertical and forward work as well as total internal, external and mechanical work are the most reduced parameters compared to 1 g indicating extremely large effects (>0.9).

For the biomechanical parameter recovery (ability of the human body to safe energy by behaving like a pendulum-like system), especially in Martian gravity different effects and direction of changes were found ranging from small to extremely large changes depending on locomotion modes and velocities (Figure 9).

For joint kinematics only effect sizes for Lunar gravity compared to 1 g are presented and indicate reductions with extremely large effects for hip and knee range of motion using the tilted and vertical body weight support systems. For ankle range of motion, effect sizes cover the whole range from small to extremely large (Figure 7).

Most of the spatio temporal parameters showed extremely large effects in all defined gravity ranges (Figures 7–9). Examples of these effects include increased swing phase and cycle duration in Lunar gravity, increased Froude number and decreased preferred walk-to-run transition speeds in Lunar and Martian gravity. One exception for the overall extremely large reduced stride frequency was found in the study of Pavei et al. (2015) for walking in Lunar and Martian gravity (small effect) using the vertical body weight support system. In partial gravity, stride length is mostly reduced during walking (Donelan and Kram, 1997) and increased during running (Donelan and Kram, 2000; Cutuk et al., 2006) with extremely large effect sizes. Stride length data from Ivanenko et al. (2002) indicate moderate to extremely large effects in 0.25 g depending on the walking speed (Figure 8). Duty Factor is reduced in partial gravity compared to 1 g but beside extremely large effects, also moderate effects are presented by Donelan and Kram (1997) for walking at fixed Froude numbers in 0.25 g.

Ground reaction forces (GRF) (except relative values) and impulses are reduced in partial gravity compared to 1 g and involve extremely large effects. Contrary, the time to impact peak force is increased in 0.25 g and Martian gravity but again with an extremely large effect.

## Discussion

### Summary of main results

The main findings of this study were the heterogeneity of results across studies, the extremely large effect sizes within a wide range of effect sizes, the low quality of applied methodologies as well as the discovery of a significant lack of knowledge concerning long-term adaptations in partial gravity. The longest continuous exposure to partial gravity reported in one of the included studies was a period of 2 weeks, with 9.6° head-up tilt during daytime and 0° supine position during the nights (Baranov et al., 2016). The reasons for the heterogeneous findings across studies can be explained as follows: (1) The included studies reported a wide range of ages; (2) Studies were performed with both male and female participants. For example Evans et al. (2013) found different significant results in diastolic blood pressure for males and females; (3) The presentation of data varies from study to study. While some authors reported absolute values, some others reported relative values using different normalization reference values; (4) Gravity levels were inconsistent between studies because not all studies used the exact gravity levels of 0.16 g for the Moon and 0.38 for Mars; (5) Durations of partial gravity exposure varied depending on the used simulation model [e.g., 25–30 s of partial gravity exposure during parabolic flights (Aerts et al., 2012) vs. 6 h head-up tilt (Lathers et al., 1990, 1993; Lathers and Charles, 1994)]; (6) Different velocities and locomotion types or postures (e.g., walking vs. running or standing vs. sitting) were used in the different protocols. Donelan and Kram (1997, 2000) found significant different results for relative stride length at same speed depending on walking or running protocols; (7) Varying experimental conditions were reported between studies. While most of the included studies tested their participants in sport clothes, others performed their experiments with space suits, very likely leading to restrictions/alterations of movements (Spady and Krasnow, 1966; Robertson and Wortz, 1968; Spady and Harris, 1968); (8) Diverse experimental set ups were used (e.g., rolling vs. a fixed trolley for the vertical body weight support system); (9) Various partial gravity simulation models were included but all of them have certain limitations. For instance not all simulation models are suitable to expose the whole body to a partial gravity environment or to simulate realistic hemodynamic changes (e.g., vertical body weight support systems) and therefore the impact on the cardiovascular system may vary from model to model (see Section Validity of included partial gravity simulators).

In all included studies the cardiopulmonary parameters heart rate, oxygen consumption, respiratory rate, expired minute volume, (net) metabolic rate, locomotion efficiency, cost of transport and bioelectrical thoracic impedance revealed either decreasing trends or significant reductions with decreasing gravity levels. On the other hand, stroke volume seems to increase with decreasing gravity levels. For blood pressure parameters, no consistent results were found. Some studies reported increasing values (Cutuk et al., 2006; Evans et al., 2013; Kostas et al., 2014) and some other studies reported decreasing values (Cardus, 1996; Chang et al., 1996; Aerts et al., 2012; Kostas et al., 2014; Widjaja et al., 2015) or unchanged values (Schlabs et al., 2013) in response to changing gravity levels from 1 g. However, effect sizes for most of the cardiac as well as metabolic outcomes and for two exceptions concerning blood pressure parameters were extremely large.

Data obtained during the Apollo missions 11–17 reveal that during actual Lunar surface explorations mean heart rates were 90–100 beats per minute (bpm) with maximum values of 160 bpm (Kopanev and Yuganov, 1975). Metabolic rates had a total mean of 234 kilocalories per hour within a total time of 159 h of Lunar Extravehicular Activities (EVA; Waligora and Horrigan, 1975). Importantly, data from the Apollo missions have to be interpreted with caution as Apollo astronauts were restricted in their movements through their space suits, making it impossible to compare Apollo data to most of the data obtained in lab conditions.

The biomechanical data of the included studies duty factor, vertical impact loading rate, active force peaks, peak vertical and horizontal impulses, horizontal and vertical work as well as the resultant total external, internal and mechanical work per unit distance decreased significantly with decreasing gravity levels. The preferred walk-to-run transition speed revealed a decreasing trend while recovery of mechanical energy during walking, range of motion for hip and knee angles, stance phase duration, ground contact time, stride frequency and (net/normalized) vertical peak GRF mostly showed decreasing trends and partly significant reductions with decreasing gravity levels. All included studies presented increasing trends for the Froude number, vertical spring stiffness and with one exception (during walking; Sylos Labini et al., 2011) also for swing phase duration and stride length. Further, a significant increase for time to impact force peak (vertical GRF) with decreasing gravity levels was shown. For Electromyography (EMG) amplitude and activation patterns inconsistent results were reported, with studies reporting changes in all directions depending on locomotion velocity (Ivanenko et al., 2002) or no (abrupt) changes at all (Sylos Labini et al., 2011).

The largest effect sizes were associated with parameters influencing the center of mass oscillation such as internal, external and mechanical work and for GRF and impulses where extremely large effects were presented. These outcomes together with cardiac and metabolic parameters are therefore the main areas that operational guidelines and decision making need to consider. Future research should attempt to address these same issues ahead of upcoming exploration missions to minimize risks to the astronauts.

### Quality of evidence

#### Validity of included partial gravity simulators

Terrestrial partial gravity simulation models seek to simulate reduced gravity and its impact on human physiology as close as possible to the actual Lunar or Martian environment. A main problem is the lack of “real” partial gravity data and therefore, it is almost impossible to validate current partial gravity simulation models. Nevertheless, a quality appraisal of included technical principles to create partial gravity conditions was performed by the authors (see Table 4). Partial gravity as created through parabolic flights was set as a gold standard with the highest possible rating. The reason for this is that parabolic flights create partial gravity that affects all physiological systems similar to “real” partial gravity on the surface of the Moon or Mars. Obviously, considering the very short exposure times during parabolic flights, the model validity must only refer to immediate physiological adaptations. Slow reacting systems cannot be studied using parabolic flights and require different models. Thus, considering the aim of the present quality appraisal all methods were rated as per how accurate they can mimic the effects of partial gravity for relevant physiological and biomechanical categories. The ratings are based on the advantages and limitations of the included simulation models and were performed in agreement with physiological and biomechanical experts from ESA's Space Medicine Office and from the German Sport University.

As shown in Table 4, all models are suitable to manipulate GRF very accurately whereas cardiovascular responses are dependent on the posture of the body (e.g., degree of body tilt) or systems that promote fluid shifts (e.g., lower body positive pressure). Kinematics were only rated as quite accurate because movements are influenced by the set-up of the included simulation systems which may limit natural friction-free movements (e.g., using rubber cords or exoskeletons). As GRF can be mimicked very precisely and kinematics are quite accurate, biomechanics can be investigated in all included simulation models quite accurately. This includes muscle activation patterns with the one exception that compared to the suspension systems, lower body positive pressure treadmills are probably closer to “real” partial gravity. This is because of the free moving limbs when walking/running on a lower body positive pressure treadmill and thus both the stance and the swinging legs are exposed to partial gravity at all times. Respiratory and metabolic properties were rated as quite accurate but not perfect due to the movement constraining nature of all partial gravity simulators.

Different simulation models affect different physiological systems in different ways. This may explain, why for cardiopulmonary outcomes within this review, mainly partial gravity parabolic flights, head-up tilt or lower body positive pressure models were used. In agreement with the rating performed by Chappell and Klaus (2013) the vertical body weight support system was only used to investigate metabolic or respiratory changes but not for cardiovascular properties. Biomechanical outcomes within this review were mainly investigated using a vertical body weight support system as it is a valid method to reduce GRF while almost preserving natural movements. Results from different models may therefore vary and comparisons between models should be made with caution.

#### Quality of statistical analyses of included studies

Statistical analyses of included studies were in many cases deficient or not performed at all. The reason for this is probably the often very limited number of participants without normal distributed data. Therefore, the sample sizes probably failed to provide adequate power to draw conclusions about all outcome parameters using traditional significance testing. Furthermore, almost all studies had no separate control group and several studies did not involve both genders equally. Unfortunately, in most of the included studies means and standard deviations for the experimental as well as for control conditions were not presented and had to be requested. Hence, the authors of this article were limited by the data available and in some cases only visual inspection of figures was possible. Additionally, statistics sometimes failed to address the research questions of this study and therefore some of the presented *p*-values could not be used. In other cases no *post-hoc* tests were performed indicating the direction of changes or the significance level alpha was set to a rather liberal alpha = 0.1 (Lathers et al., 1990, 1993; Lathers and Charles, 1994).

### Overall completeness and applicability of evidence

Not all of the outcomes defined in the PICOS have been investigated in the included studies. Some parameters such as the arterio-venous oxygen difference are missing and diverse respiratory parameters (except of oxygen consumption) are very sparse being only investigated in one study (Robertson and Wortz, 1968). The same can be said for venous hemodynamics. Morphological parameters such as fiber type composition, muscle fiber length, physiological and anatomical cross sectional areas, muscle pennation angles, tendon function and material properties as well as bone mineral density are completely missing but are important indicators for physiological deconditioning and very relevant for space flight operations. Obviously, changes of these parameters can only be investigated during long-term exposure to partial gravity, and as already mentioned there is a lack of long-term partial gravity studies. Furthermore, muscle force, angular velocities and joint torques have not been investigated but are important measures for the mechanical strain in the musculoskeletal system. Of all included technical principles to simulate partial gravity only supine suspension systems are missing. Despite a lack of important outcome parameters, the 43 included studies were overall sufficient to address the objectives of this review, even if in some cases it was necessary to “read between the lines” and to filter relevant results for this systematic review.

### Potential bias in the review process

The strict methodology of this review with clearly defined inclusion criteria as well as a comprehensive search strategy minimized the potential for bias.

The literature research was hindered by the design of some databases. Databases such as the Erasmus Experiment Archive of ESA do not offer “advanced search methods” and had therefore to be searched manually. Obviously, this increases the risk of failing to include relevant studies. Furthermore, misleading or wrong terminology may have led to the undesired exclusion of relevant studies. For example “reduced gravity” was often used as a synonym for μg and not for partial gravity.

In some cases the authors had to obtain data from figures instead of numeric tables (e.g., from conference presentation slides of Cowley et al., 2015). Possibly, this could have introduced a potential bias, as the measurements on figures and detection of (visual) changes are not 100% accurate and could vary from person to person. Therefore, smallest differences from control were defined as changes, even if statistically no significant results were reported. In the result Supplementary Tables 1–6, arrows without an asterisk indicate this fact and should be interpreted with caution.

### Agreements and disagreements with other studies

Due to the comprehensive literature research there are almost no experimental studies left to which the present results can be compared. Therefore, also computational models/simulations were included for this comparison to see if experimental data show similar changes in magnitude and direction as modeled data.

#### Comparison with modeled data

The kinematic model of Raichlen (2008) which predicts the effects of gravity on human locomotion matches the data as presented in this review. The author calculated, that relative stride lengths at 2 meters per second (m/s) as well as the Froude number at walk-to-run transition speed increases with decreasing gravity (Raichlen, 2008). For Froude number, the same increasing tendency was estimated from audio transcripts and video clips of Lunar EVA's as well as by the astronauts and space suit characteristics by Carr and Mcgee (2009). The latter study also found out, that wearing a spacesuit appears to lower the Froude number and the walk-to-run transition will occur at lower velocities. Therefore, they suggest the introduction of an “Apollo number” (Froude number divided by mass) to capture the effects of spacesuit self-support (Carr and Mcgee, 2009). Ackermann and van Den Bogert (2012) predicted values for different locomotion types in partial gravity through a computational simulation using a realistic musculoskeletal model. They calculated reduced vertical GRF for each gait type compared to terrestrial gravity which is in agreement with the present findings. They also predicted skipping as the preferred gait mode in Lunar gravity because their results suggest that skipping is more efficient and less fatiguing compared to walking or running (Ackermann and van Den Bogert, 2012).

Keller and Strauss (1992) predicted bone mineral density changes in partial gravity using modeled data. They predicted a weekly loss of 0.39% for bone mineral density in a Lunar- and a loss of 0.22% in a Martian gravity environment. Unfortunately, no included study of the present review investigated changes in bone mineral density. Nevertheless, it should be pointed out that this model predicted that bone mineral density loss will not be prevented in a partial gravity environment. Provided the mathematical modeling is accurate, it appears that planetary stay times could be extended from ~100 weeks on Moon to 3 years on Mars (based on the assumption that until then a reduction of 66% in strength is attained) before a weakened skeleton could create serious hazards during the stresses of re-entry and returning to terrestrial gravity (Keller and Strauss, 1992). Obviously, the latter assumption represents a pure scientific point of view and is certainly not in line with medical operations guidelines.

#### Comparison with other review articles

There are only very few reviews about partial gravity research but none of them used such a systematic and comprehensive search strategy as this study. Davis and Cavanagh (1993) focused on biomedical issues related to human locomotion in partial gravity. They summarized (using data from up to 3 included experiments and calculations from a ballistic walking model) that swing phase as well as stance phase duration is increasing with decreasing gravity whereas cadence and walking velocity is decreasing. The latter also affected peak vertical GRF which were decreased in reduced gravity (Davis and Cavanagh, 1993). This is in agreement with the findings of the present review with the exception of stance phase duration which was reported to decrease in all of the included studies (Ivanenko et al., 2002, 2011; Sylos Labini et al., 2011; Cowley et al., 2015).

Shavelson (1968) summarized findings of different studies on metabolic rate and concluded that in four out of five studies metabolic rate is decreasing with decreasing gravity unless high mechanical work and external forces are required (Shavelson, 1968). Their findings are in agreement with the present results.

The review article of Sylos-Labini et al. (2014) included mainly studies which have been investigated in this systematic review but without a comparable systematic approach and not fully in agreement with the present outcome parameters as defined in the PICOS. The present outcomes cover a wider spectrum of parameters which are considered as operationally relevant by ESA's Space Medicine Office, in particular for future planetary explorations. Finally, the review article of Sylos-Labini et al. (2014) did not cover the whole range of available literature about biomechanics in partial gravity.

#### Studies that “slipped through”

Despite the fact that we have applied a comprehensive strategy, there were a few relevant studies that escaped our search. This could have been for the following reasons: Studies were published after the period of this literature search or studies did not cover included keywords or were not listed in included databases. These studies were either found through random online searches or were cross-referenced in one of the included studies. Data of these studies were not extracted for the present review however the findings of these studies and the findings of this systematic review are compared in the following. The study of Ruckstuhl et al. (2009) that compared gait parameters and heart rate as measured using lower body positive pressure or vertical body weight support (33% BW) during different walking speeds (0.5–1.2 m/s) was not found during the present research process. Nevertheless, it fits with the defined PICOS and results are in agreement with findings of this systematic review. In their study, heart rate, stride frequency and duty factor decreased significantly with decreasing gravity levels. For Martian gravity, normalized stride length was not found in the present results but Ruckstuhl et al. (2009) presented a significant reduction in their results. For leg angle at touch down they showed a significant increase compared to terrestrial gravity. Further, Ruckstuhl et al. (2009) compared lower body positive pressure and vertical body weight support systems and found no significant differences for the gait parameters but did for heart rate (Ruckstuhl et al., 2009). This is in agreement with the conclusions of the present quality appraisal of included technical principles to simulate partial gravity (see Table 4).

One of the most recent studies about musculoskeletal changes due to partial gravity exposure is the study of Ritzmann et al. (2016) which is not included in this systematic review because it was not published during the time of the present literature search. The authors measured biomechanical parameters of a bouncing movement (often referred to as skipping) during a partial gravity parabolic flight (Mars and Moon parabolas). Their results show a reduction of peak vertical GRF, rate of force development and vertical impulse with decreasing gravity (Ritzmann et al., 2016). This is in agreement with the results presented in this systematic review whereas joint angles and EMG can hardly be compared to the present results because bouncing movements and normal walking or running are quite different. The main conclusion of the study by Ritzmann et al. (2016) was that subjects are able to keep their motor control patterns. They suggest that muscle activity in changed gravity environments can be anticipated (shown in a decline in activation amplitudes before touchdown) and resulting muscle forces can be properly adjusted.

### Relationship and interplay between biomechanical and cardiopulmonary outcome parameters

**Figure 10:**
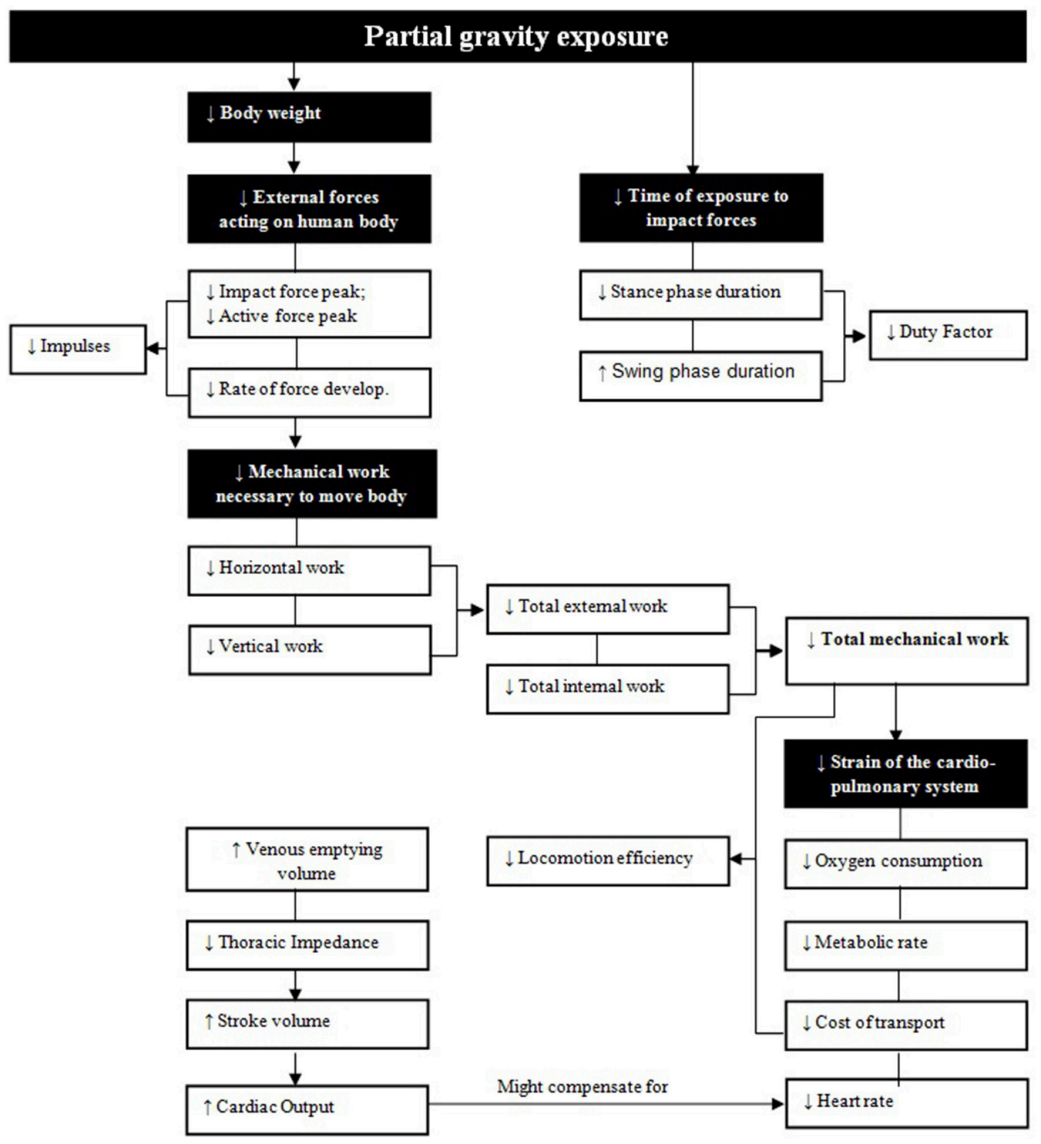
Interaction of cardiopulmonary and biomechanical parameters. Black boxes represent physiological main factors of exposure to partial gravity while in the white boxes underlying outcome parameters are presented.

Exposure to partial gravity reduces body weight and therefore external forces acting on the human body (Figure 10). This can be seen in the reduced vertical GRF with a reduced first impact and second active force peak as well as a reduced rate of force development. As the area under the force-time curve becomes smaller, also impulses are reduced. Additionally, the time of exposure to impact forces becomes less as stance phase duration, ground contact times and duty factor decrease. As a consequence, it is likely that the reduced mechanical stimuli (supported by extremely large effects) associated with walking and running in Moon and Mars gravity conditions will not be sufficient to fully maintain terrestrially optimal bone mineral density and muscle mass in the long-term. Further, due to partial gravity-induced mechanical unloading, the mechanical work that is necessary to move the body becomes less. In the present data this becomes apparent in the reduction of horizontal and vertical work per distance, resulting in a reduced total external work. Together with a reduced total internal work necessary to rotate and accelerate limbs, the total mechanical work is decreased in partial gravity environments as can be seen in the extremely large effects. Most likely this explains the reduced load on the cardiopulmonary system in reduced gravity. For instance, heart rate and oxygen consumption correlate with work performance and therefore it does not surprise that these parameters are decreased in partial gravity. Rates of oxygen consumption and carbon dioxide production are measured to estimate (net) metabolic rates and as oxygen consumption decreases it seems to be logical that metabolic rate also decreases with decreasing gravity levels. If the mass specific metabolic rate is divided by speed, the net cost of transport can be calculated. The relative metabolic cost of transport at similar velocities is therefore also reduced in partial gravity environments. This means that less physical effort is necessary to move the body. As locomotion efficiency (defined as the total mechanical work divided by cost of transport) is reduced as well as both total mechanical work and cost of transport are reduced in partial gravity, total mechanical work must be reduced by a greater extent than cost of transport. Under consideration that partial gravity leads to a thoracic fluid shift, as indicated by the reduced thoracic impedance and the increased venous emptying volume, a higher blood volume in the region of the heart is very likely to lead to an increase in stroke volume. If stroke volume increases more than heart rate decreases, cardiac output must be increased (as found in the results) and might compensate for the reduction in heart rate.

### Relevance for future human space explorations and countermeasure developments

#### Anticipated consequences of reduced mechanical and metabolic stimuli in partial gravity

As described above, reduced impact forces due to partial unloading may lead to reductions of the work necessary to move the human body. This in turn may have detrimental long-term effects on the cardiopulmonary system, likely resulting in a loss of work performance capacity. Due to a reduction of important mechanical and metabolic stimuli the body is set into a “fake” resting state, affecting physiological systems and in the worst case resulting in physiological degeneration beyond (long-term-) mission threatening levels. It is very important that EVA's of the astronauts are completed without exhaustion and that their physical well-being is maintained for reasons of health, safety and mission success.

From an operational perspective it would be highly desirable to know minimum thresholds and exposure times to certain gravity levels that are needed to maintain relevant physiological systems (Horneck et al., 2003; Goswami et al., 2012). These systems will presumably react differently to similar gravity levels and therefore it is very unlikely that one minimum gravity threshold is sufficient to maintain all physiological systems equally. It can be anticipated from linear regression analyses that for some systems the lack of sufficient mechanical physiological stimuli becomes less severe as gravity increases. Some studies showed that there is a strong correlation between heart rate (Schlabs et al., 2013), oxygen consumption (Schlabs et al., 2013), (net) metabolic rate (Farley and McMahon, 1992; Teunissen et al., 2007), peak vertical ground reaction force (Ivanenko et al., 2002; Schlabs et al., 2013) and the simulated gravity levels in the range between 1 g and μg (with *R*^2^ > 0.88 for all tested correlations). Therefore, exposure to Moon and Mars gravities might be less severe compared to physiological deconditioning as experienced in μg.

#### Requirements for exercise countermeasure concepts in partial gravity

To compensate for the anticipated loss in performance capacity some form of supplementary exercise will most likely be required. The slogan “use it or lose it” describes the adaptation process in a very simple way (Corcoran, 1991), and may also be applied to partial gravity environments.

As pointed out, reduced external forces acting on the body seem to be a main problem because a reduction of mechanical stimuli could also account for a reduction in metabolic stimuli. Therefore, exercise countermeasures should provide an individual, comprehensive training and especially focus on applying Earth-like GRF.

GRF can be modified/increased through increased locomotion velocity (Davis and Cavanagh, 1993), external applied horizontal forces (Chang et al., 2001) or reactive jumps (Kramer et al., 2010). Davis and Cavanagh (1993) provide the example that running at 4 m/s at 80% body weight creates the same magnitude of vertical GRF as running at 2.9 m/s at 100% body weight. Moreover, Chang et al. (2001) found out, that running at 0.38 g with 20% of additional applied horizontal forces increases impact force peaks to magnitudes equal or greater than those observed during running at Earth gravity. Furthermore, jumps induce high impact forces and internal muscle forces that are necessary for the deformation of bone and thus provide an osteo- and muscle-protective stimulus (Rittweger et al., 2000; Ebben et al., 2010). At the same time, plyometric exercise can be very exhaustive and therefore high workload- or high intensity interval protocols could induce cardiovascular responses, preventing cardiovascular deconditioning (Arazi et al., 2012, 2014). The workgroup of Kramer (University of Konstanz, Germany) invented a new sledge jump system which allows after some practice almost natural reactive jumps in reduced gravity (Kramer et al., 2010, 2012). This seems to be a promising countermeasure as it provides myogenic as well as osteogenic stimuli while only short exercise durations are necessary.

Unfortunately, there is no “one-fits-all”- strain level to maintain bone mineral density because it is affected by skeletal location and other systemic factors such as age, gender, and genetic background (Ruff et al., 2006). This can also be said for muscle mass, as the active tension required to induce hypertrophy or prevent atrophy is very likely to vary as it is subordinate to the complex “response matrix” of the respective subject (Toigo and Boutellier, 2006). Nevertheless, Frost (2004) refers to a bone's genetically determined modeling threshold strain range (1,000–1,500 microstrain; ~2 kg/mm^2^), within and above which formation of new bone exceeds resorption of bone mineral (Frost, 2004). Therefore, exercise countermeasures should aim at exposing the bones to up to 1,000 microstrain to at least maintain its strength. The study results of Peterman et al. (2001) reveal that bone strain magnitudes in the distal tibia are linearly related to GRF (*R*^2^ > 0.7) (Peterman et al., 2001) which supports the authors suggestion that exercise countermeasures should focus on applying Earth like GRF as experienced during running and jumping.

## Conclusion and outlook

This systematic review provides insights into the current state of research about human biomechanical and cardiopulmonary responses to partial gravity exposure. The synthesized results presented here suggest a lack of sufficient metabolic and mechanical stimuli when humans are exposed to partial gravity as can be seen in the extremely large effects of most of the presented outcomes. It can be anticipated that partial gravity environments as present on the Moon or on Mars are not sufficient to preserve all physiological systems to a 1 g standard if not addressed through adequate countermeasures. Therefore, to maintain astronaut's health, safety and performance capacity smart and evidence-based exercise countermeasure systems are needed. The main goal of these systems should be to re-create Earth-like GRF. Considering the smaller habitat/vehicle size to be used in future exploration missions, countermeasure devices should be as compact as possible but still target the musculoskeletal and cardiopulmonary systems equally. Bulky exercise machines as currently used on the ISS (e.g., ARED, cycle ergometer or treadmills) will not be an option for these missions.

The methodological quality of the vast majority of the available/included studies is too low to generate a compeling evidence. Future research is needed and should address physiological long-term effects of partial gravity exposure. Moreover, future studies should help defining minimal gravity thresholds and exposure times needed to maintain relevant physiological systems.

## Author contributions

All authors listed have made a substantial, direct and intellectual contribution to the work, and approved it for publication.

### Conflict of interest statement

The authors declare that the research was conducted in the absence of any commercial or financial relationships that could be construed as a potential conflict of interest.

## Supplementary material

Supplementary Table 1 Cardiopulmonary changes in Lunar gravity.

Supplementary Table 2 Cardiopulmonary changes in 0.25 g.

Supplementary Table 3 Cardiopulmonary changes in Martian gravity.

Supplementary Table 4 Biomechanical changes in Lunar gravity.

Supplementary Table 5 Biomechanical changes in 0.25 g.

Supplementary Table 6 Biomechanical changes in Martian gravity.

Supplementary Tables 1–6 In the first row, information about authors, their used simulation model (HDT: head-down tilt), posture or locomotion (^w^ walking; ^r^ running; ^s^ skipping; ^h^ hopping; ^PTS^ moving at preferred walk-to-run transition speed) during the intervention as well as number of participants and control conditions are presented. Terrestrial gravity as a control condition indicates that changes from 1 g to partial gravity have been considered, whereas μg as a control indicates that changes from μg to partial gravity have been considered. In the left column the different outcome parameters are listed and assigned to categories. Arrows indicate an increasing (↑), decreasing (↓), or stable (→) trend. Asterisks (^*^) indicate significant differences from control conditions (*P* < 0.05) and hashtags (^#^) refer to partly significant differences.

## Abbreviations

μg: Microgravity
ARED: Advanced Resistive Exercise Device
bpm: Beats per Minute
BW: Body Weight
BWS: Body Weight Support (System)
RCT: Randomized Controlled Trial
CoM: Center of Mass
DLR: German Aerospace Centre
EMG: Electromyography
ESA: European Space Agency
EVA: Extravehicular Activity
g: Gravitational Acceleration
GRF: Ground Reaction Force
^h^: Hopping
HUT: Head-Up Tilt
ISS: International Space Station
LBPP: Lower Body Positive Pressure
MeSH: Medical Subject Headings
m/s: Meter per Second
NASA: National Aeronautics and Space Administration
pg: Partial Gravity
PICOS: Eligibility Criteria (Population, Intervention, Control, Outcome, Study Type
PRISMA: Preferred Reporting Items for Systematic Reviews and Meta-Analyses
^PTS^: Preferred Walk-to-Run Transition Speed
^r^: Running
^s^: Skipping
^w^: Walking.
