# Supplementary material for: Human Biomechanical and Cardiopulmonary Responses to Partial Gravity – A Systematic Review
Source: Front Physiol. 2017 Aug 15;8:583. doi: 10.3389/fphys.2017.00583 (PMC5559498; doi:10.3389/fphys.2017.00583)
Supplement: Supplementary Table 2 — Cardiopulmonary changes in 0.25 g. [file Table2.pdf]

		Farley & McMahon 1992	Fox et al. 1975	Grabowski et al. 2005	Teunissen et al. 2007
	**Simulation model**	**vertical BWS**	**centrifugation**	**vertical BWS**	**vertical BWS**
	**Posture/Locomotion**	**1 m/s** [w]<br>**3 m/s** [r]	**0.9-1.8 m/s** [w]	**1.25 m/s** [w]	**3 m/s** [r]
	**Number of participants**	**n = 4**	**n = 4**	**n = 10**	**n = 10**
	**Control condition**	1g	1g	1g	1g
Car-diac	**Heart rate** [bpm]		↓*		
Respi-ratory	**Relative oxygen consumption** [ml/kg/min]		↓*		
Metabolic	**Net metabolic rate** [W/kg^{-1}]	↓*		↓*	↓*
	**Cost of transport** [J/kg/min]	↓*			
	**Mechanical efficiency** [mlVO2/kg/min]		→		
