# Supplementary material for: Human Biomechanical and Cardiopulmonary Responses to Partial Gravity – A Systematic Review
Source: Front Physiol. 2017 Aug 15;8:583. doi: 10.3389/fphys.2017.00583 (PMC5559498; doi:10.3389/fphys.2017.00583)
Supplement: Supplementary Table 3 — Cardiopulmonary changes in Martian gravity. [file Table3.pdf]

		Aerts et al. 2012		Cardus 1996	Chang et al. 1996		Cutuk et al. 2006	Evans et al. 2013		Kostas et al. 2014		Lathers et al. 1990, 93, 94	Pavei et al. 2015	Pavei & Minetti 2015	Schlabs et al. 2013		Widjaja et al. 2015	
	**Simulation model**	**partial g parabolic flight**		**centrifugation**	**HUT**		**LBPP**	**LBPP**		**LBPP, HUT**		**HUT**	**vertical BWS**	**vertical BWS**	**LBPP**		**partial g parabolic flight**	
	**Posture/Locomotion**	**sitting**		**supine**	**semi-supine**		**standing, 1.34 m/s** ^{w}	**standing**		**standing, semi-supine**		**semi-supine**	**0.83-3.61 m/s** $^{w, s, r}$	**0.56-2.5 m/s** $^{w, r, s, h}$	**standing, 0.9 m/s** ^{w}		**sitting**	
	**Number of participants**	**n= 6**		**n = 4**	**n = 12**		**n = 15**	**n = 14**		**n = 21**		**n = 6**	**n = 13**	**n = 6**	**n = 12**		**n = 14**	
	**Control condition**	1g	µg	1g	1g	µg	1g	1g	µg	1g	µg	µg	1g	1g	1g	µg	1g	µg
**Cardiac**	**Heart rate** [bpm]			↓			↓*	↓*	↑*	↓*	↑ LBPP<br>→ HUT	↓ 2h<br>→ 4h<br>↑ 5h, 6h			↓	↑	↓	↑
	**NN interval, meanRR** [ms]	↓	↓														↑*	↑
	**Heart rate variability (SDNN, stdRR)** [ms]	↑	↓														↑	↓
	**Stroke volume** [ml]			↑				↑*	↓*	↑*	↓*	↓ 2h, 4h, 5h, 6h						
	**Cardiac output** [l/min]			↑						↑	↓	↓ 2h, 4h, 5h, 6h						
	**Left ventricular systolic volumes** [ml]											↓ 2h, 4h, 5h, 6h						
	**Left ventricular diastolic volumes** [ml]											↓ 2h, 4h, 5h, 6h						
**Blood Pressure**	**Systolic blood pressure** [mmHg]	↓*	↓	↓			↑			↑		↑ 2h, 4h, 5h, 6h					↓	↓
	**Diastolic blood pressure** [mmHg]	↓*	↓	↓			↑	↑	↑#			↑ 2h, 4h, 5h, 6h					↓	↓
	**Mean arterial blood pressure** [mmHg]				↓	↑	↑			↑	↑	→ 2h<br>↑ 4h, 5h, 6h			→		↓	
	**blood pressure ramps** [%]							↓	↓									
	**Total peripheral resistance** [mmHg/l/min]							↑	↑*	↓	↑	↑ 2h, 4h, 5h, 6h						
**Haemodynamics**	**Blood flow velocity** [cm/s]				↑ foot sole	↓ foot sole	↑ middle cerebral artery											
	**Bioelectrical thoracic impedance** [Ω]			↓				↓	↑*	↓	↑ LBPP<br>↓ HUT							
	**Bioelectrical abdominal impedance** [Ω]							↓	↓*	↓ LBPP<br>↑ HUT	↓							
**Respira-tory**	**Oxygen consumption** [ml/kg/min]														↓	↑		
**Metabolic**	**Locomotion efficiency**												↓*	↓				
	**Cost of transport** [J/kg/min]												↓#	↓#				
