# Supplementary material for: Human Biomechanical and Cardiopulmonary Responses to Partial Gravity – A Systematic Review
Source: Front Physiol. 2017 Aug 15;8:583. doi: 10.3389/fphys.2017.00583 (PMC5559498; doi:10.3389/fphys.2017.00583)
Supplement: Supplementary Table 4 — Biomechanical changes in Lunar gravity. [file Table4.pdf]

		Cavanagh et al. 2013	Cutuk et al. 2006	De Witt et al. 2014	Cowley et al. 2015	He et al. 1991	Ivanenko et al. 2011	Kram et al. 1997	Sylos Labini et al. 2011	Pavei et al. 2015	Pavei & Minetti 2015	Schlabs et al. 2013	Sylos-Labini et al. 2013	Spady & Harris 1968	Spady & Krasnow 1966
	**Simulation model**	**HUT**	**LBPP**	**partial g parabolic flight, vertical BWS**	**partial g parabolic flight, vertical BWS**	**vertical BWS**	**vertical BWS, tilted BWS**	**vertical BWS**	**vertical BWS, tilted BWS**	**vertical BWS**	**vertical BWS**	**LBPP**	**vertical BWS, tilted BWS**	**tilted BWS** with pressurized suit	**tilted BWS** with pressure suit
	**Posture/Locomotion**	**semi-supine, sitting**	**1.34 m/s [w] 2.68 m/s [r]**	**Ø 1.42 m/s PTS**	**0.8 m/s [w]**	**3 m/s [r]**	**individual [PTS]**	**Ø 0.97-1.18 m/s [PTS]**	**individual [PTS]**	**0.83-3.61 m/s [w, s, r]**	**0.56-2.5 m/s [w, r, s, h]**	**standing, 0.6-0.9 m/s [w]**	**0.6 m/s [w], 1.4 m/s [r]**	**0.8 m/s slow[w], 1.4 m/s norm.[w], up to 4.7 m/s [r]**	**0.5-1.5 m/s [w], 1.1-3.7 m/s [r]**
	**Number of participants**	**n = 5**	**n = 15**	**n = 8**	**n = 15**	**n = 4**	**n = 8**	**n = 9**	**n = 6**	**n = 13**	**n = 6**	**n = 12**	**n = 7**	**n = 2**	**n = 5**
	**Control condition**	1g	1g	1g	1g	1g	1g	1g	1g	1g	1g	1g	1g	1g	1g
	**Muscle volume quadriceps**	↓													
**CoM Oscillation**	**Total external work** [J·kg^{-1}·stride^{-1}]									↓*	↓*				
	**Total internal work** [J·kg^{-1}·stride^{-1}]									↓*	↓*				
	**Total mechanical work** [J·kg^{-1}·m^{-1}]									↓*	↓*				
	**Recovery of mechanical energy** [%]									↓ walk. ↑ skip., run					
	**Vertical displacement of CoM** [cm/stride]					↓	vert. hip displacem.: no abrupt changes			↓					
	**Vertical velocity of CoM** [m/s]					→									
**Joint Kinematics**	**Hip angle at stance** [°]				→									↑ flex.	
	**Knee angle at stance** [°]				↑ flex.									slow walk.: ↑ flex. normal walk.: →	
	**Ankle angle at stance** [°]				↑ dorsalflex.									→	
	**Touch down angle leg** [°]					↓	limb axis angle: no abrupt changes								
	**Hip range of motion** [°]				↓								↓*	→	
	**Knee range of motion** [°]		↓		↓								↓*		
	**Ankle range of motion** [°]		↑ walk. ↓ run		↓										
	**Ankle range of motion at 2km/h** [°]												→ (vert. BWS) ↑ (tilt. BWS)		
	**Ankle range of motion at 5km/h** [°]												↓* (vert. BWS) ↑ (tilted BWS)		
**Spatio Temporal Parameters**	**Froude number**			↑			↑	↑		↑					
	**Cycle duration at 2 km/h** [s]												↑* (tilted BWS,) → (vert. BWS)		
	**Cycle duration at 5 km/h** [s]												↑*		
	**Ground contact time** [s]					↓					→				
	**Stance phase duration** [% cycle], [s]				↓		↓		↓						
	**Swing phase duration** [% cycle], [s]				↑		↑*		↓ walk. ↑ run.				rel. ↑[#]		
	**Flight phase duration** [% cycle], [s]										↑ hopping				
	**Frequency** [Hz]				↓	↓				→ walk. ↓* run., skip.	↓*			↓	
	**Stride length** [m], [cm]		↑		↑									↑	~ normal, unpress. ↑* press.
	**Preferred transition speed** [m/s]			↓			↓	↓	↓						
	**Walking velocity** [m/s]														↓ normal, unpress ↑* press.
**GRF**	**Vert. peak ground reaction force** [N]		net ↓*		normalized: ↓	↓						normalized: ↓			
**Joint Kinetics**	**Leg stiffness** [kN/m]					→									
	**Vertical stiffness** [kN/m]					↑									
**EMG**	**EMG & H-reflex pattern**								no abrupt changes						
