# Supplementary material for: Human Biomechanical and Cardiopulmonary Responses to Partial Gravity – A Systematic Review
Source: Front Physiol. 2017 Aug 15;8:583. doi: 10.3389/fphys.2017.00583 (PMC5559498; doi:10.3389/fphys.2017.00583)
Supplement: Supplementary Table 5 — Biomechanical changes in 0.25 g. [file Table5.pdf]

		Chang et al. 2001	Donelan & Kram 1997	Donelan & Kram 2000	Farley & McMahon 1992	Ferris et al. 2001	Griffin et al. 1999	Ivanenko et al. 2002	Teunissen et al. 2007
	**Simulation model**	**vertical BWS**	**vertical BWS**	**vertical BWS**	**vertical BWS**	**vertical BWS**	**vertical BWS**	**vertical BWS**	**vertical BWS**
	**Posture/Locomotion**	**3 m/s ^{r}**	**0.25-1 m/s ^{w}**	**2-5 m/s ^{r}**	**1 m/s ^{w}**<br>**3 m/s ^{r}**	**1.25 m/s ^{w}**<br>**3 m/s ^{r}**	**1 m/s ^{w}**	**0.2-1.4 m/s ^{w}**	**3 m/s ^{r}**
	**Number of participants**	**n = 8**	**n = 10**	**n = 10**	**n = 4**	**n = 8**	**n = 6**	**n = 8**	**n = 10**
	**Control condition**	1g	1g	1g	1g	1g	1g	1g	1g
**CoM Oscillation**	**Horizontal work** [J·kg^{-1}·stride^{-1}]						↓*		
	**Vertical work** [J·kg^{-1}·stride^{-1}]						↓*		
	**$W_{CoM,f}/W_{CoM,v}$**						↑		
	**Total external work** [J·kg^{-1}·stride^{-1}]						↓*		
	**Horizontal kinetic energy fluctuation** [J]						↓		
	**Gravitational potential energy fluctuation** [J]						↓		
	**Total mechanical energy fluctuation** [J]						↓		
	**Recovery of mechanical energy** [%]						↓*		
	**Vertical displacement of CoM** [cm/stride]						↑		
**Joint Kinematics**	**Thigh elevation angle** [°]							↑*	
	**Shank elevation angle** [°]							↑*	
	**Ankle elevation angle** [°]							↑*	
	**Ankle angle during walking** [°]					↓* dorsi-flex.<br>↑ plantar-flex.			
	**Ankle angle during running** [°]					↓* dorsi-flex.<br>↓ plantar-flex.			
**Spatio Temporal Parameters**	**Duty factor**	↓*							↓*
	**Duty factor at same speed**		↓*	↓*					
	**Duty factor at same Froude nr.**		↓*	↓*					
	**Ground contact time** [s]				→ walk. ↓ run.				↓*
	**Stance phase duration** [% cycle], [s]							↓*	
	**Flight phase duration** [% cycle], [s]								↑*
	**Frequency** [Hz]	↓*				↓ walk. ↓* run.			↓*
	**Stride length** [m], [cm]							↓*	
	**Relative stride length at same speed** [cm]		↓*	↑*					
	**Relative stride length at same Froude nr.** [cm]		↓*	↓*					
**GRF**	**Vertical peak ground reaction force** [N]	↓*						↓	↓*
	**Rel. vertical peak ground reaction force at same speed** [N/kg]			↑*					
	**Rel. vertical peak ground reaction force at same Froude nr.** [N/kg]			↑*					
	**Horizontal peak ground reaction force** [N]								↓* braking<br>↓* propulsive
	**Vertical impact loading rate** [kN/s]	↓*							
	**Time to impact force peak** [ms]	↑*							
	**Active force peaks** [N]	↓*							
	**Peak vertical impulse** [N s]								↓*
	**Peak horizontal impulse** [N s]								↓*
**Joint Kinetics**	**Rel. peak leg stiffness at same Fr. nr.** [kN/m]			↓*					
	**Rel. peak leg stiffness at same speed** [kN/m]			↓*					
**EMG**	**EMG amplitude M. vastus lateralis**					→ walk. ↓* run.		→ 0,2-0.3 m/s<br>↑* 0.6-0.8 m/s<br>↓* 1.4 m/s	
	**EMG amplitude M. triceps surae**					↓*		↓*	
	**EMG amplitude M. tibialis anterior**					→		→ 0,2-0.3 m/s<br>↑* 0.6-0.8 m/s<br>↓* 1.4 m/s	
	**EMG amplitude M. biceps femoris**							↑*	
	**EMG amplitude M. rectus femoris**							→ 0,2-0.3 m/s<br>↑* 0.6-0.8 m/s<br>↓* 1.4 m/s	
	**EMG amplitude M. gluteus maximus**							↓*	
	**Max. M-wave amplitude for M. soleus** [% of standing value]					→			
	**H-reflex amplitude for M. soleus** [% of max. M-wave]					↓			
	**EMG & H-reflex pattern**					→			
