# Supplementary material for: Human Biomechanical and Cardiopulmonary Responses to Partial Gravity – A Systematic Review
Source: Front Physiol. 2017 Aug 15;8:583. doi: 10.3389/fphys.2017.00583 (PMC5559498; doi:10.3389/fphys.2017.00583)
Supplement: Supplementary Table 6 — Biomechanical changes in Martian gravity. [file Table6.pdf]

		Cavagna et al. 2000	Chang et al. 2001	He et al. 1991	Ivanenko et al. 2011	Kram et al. 1997	Sylos Labini et al. 2011	Pavei et al. 2015	Pavei & Minetti 2015	Schlabs et al. 2013
	**Simulation model**	**partial g parabolic flight**	**vertical BWS**	**vertical BWS**	**vertical BWS, tilted BWS**	**vertical BWS**	**vertical BWS, tilted BWS**	**vertical BWS**	**vertical BWS**	**LBPP**
	**Posture/Locomotion**	**0.4-1.5 m/s ^{w}**	**3 m/s ^{r}**	**3 m/s ^{r}**	**individual ^{PTS}**	**Ø 1.39 m/s ^{PTS}**	**individual ^{PTS}**	**0.83-3.61 m/s $_{w, s, r}$**	**0.56-2.5 m/s $_{w, r, s, h}$**	**standing, 0.6-0.9 m/s ^{w}**
	**Number of participants**	**n = 3**	**n = 8**	**n = 4**	**n = 8**	**n = 9**	**n = 6**	**n = 13**	**n = 6**	**n = 12**
	**Control condition**	1g	1g	1g	1g	1g	1g	1g	1g	1g
**CoM Oscillation**	**Horizontal work** [J·kg^{-1}·stride^{-1}]	↓*								
	**Vertical work** [J·kg^{-1}·stride^{-1}]	↓*								
	**Total external work** [J·kg^{-1}·stride^{-1}]	↓*						↓*	↓*	
	**Total internal work** [J·kg^{-1}·stride^{-1}]							↓*	↓*	
	**Total mechanical work** [J·kg^{-1}·m^{-1}]							↓*	↓*	
	**Recovery of mechanical energy** [%]	↓*						↓ walk. ↑ skip., run		
	**Vertical displacement of CoM** [cm/stride]			→	vert. hip displacem.: no abrupt changes			↓		
	**Vertical velocity of CoM** [m/s]			↑						
	**Touch down angle leg** [°]			↓	limb axis angle: no abrupt changes					
**Spatio Temporal Parameters**	**Froude number**				↑	↑		↑		
	**Duty factor**		↓*							
	**Ground contact time** [s]			↓					→	
	**Stance phase duration** [% cycle], [s]				↓		↓			
	**Swing phase duration** [% cycle], [s]				↑*		↓walk. ↑run.			
	**Flight phase duration** [% cycle], [s]								↑ hopping	
	**Frequency** [Hz]		↓*	↓				→ walk.<br>↓* run., skip.	↓*	
	**Preferred transition speed** [m/s]				↓	↓	↓			
**GRF**	**Vertical peak ground reaction force** [N]		↓*	↓						normalized: ↓
	**Vertical impact loading rate** [kN/s]		↓*							
	**Time to impact force peak** [ms]		↑*							
	**Impact force peaks** [N]		↓*							
**Joint kinetics**	**Leg stiffness** [kN/m]			→						
	**Vertical stiffness** [kN/m]			↑						
**EMG**	**EMG & H-reflex pattern**						no abrupt changes			
