# Supplementary material for: Human Biomechanical and Cardiopulmonary Responses to Partial Gravity – A Systematic Review
Source: Front Physiol. 2017 Aug 15;8:583. doi: 10.3389/fphys.2017.00583 (PMC5559498; doi:10.3389/fphys.2017.00583)
Supplement: Supplementary Table 1 — Cardiopulmonary changes in Lunar gravity. [file Table1.pdf]

| | | Aerts et al. 2012 | | Baranov et al. 2016 | Chang et al. 1996 | | Cutuk et al. 2006 | Evans et al. 2013 | | Kostas et al. 2014 | | Lathers et al. 1990, 1993, 1994 | Louisy et al. 1994 | | Pavei et al. 2015 | Pavei & Minetti 2015 | Pavy-Le Traon et al. 1997 | Robertson & Wortz 1968 | Schlabs et al. 2013 | | Widjaja et al. 2015 | | Waligora & Horrigan 1975 | Kopanev & Yuganov 1975 | Berry 1974 | Berry 1969 | Berry 1970 |
|---|---|---|---|---|---|---|---|---|---|---|---|---|---|---|---|---|---|---|---|---|---|---|---|---|---|---|---|
| | **Simulation model** | **partial g parabolic flight** | | **HDT-HUT** | **HUT** | | **LBPP** | **LBPP** | | **LBPP, HUT** | | **HUT** | **HDT-HUT-HDT** | | **vertical BWS** | **vertical BWS** | **HDT-HUT-HDT** | **vertical BWS, tilted BWS**<br>with spacesuit | **LBPP** | | **partial g parabolic flight** | | **Apollo mission** | **Apollo mission** | **Apollo mission** | **Apollo mission** | **Apollo mission** |
| | **Posture/Locomotion** | **sitting** | | **semi-supine** | **semi-supine** | | **standing, 1.34 m/s** $^{w}$ | **standing** | | **standing, semi-supine** | | **semi-supine** | **semi-supine** | | **0.83-3.61 m/s**<br>w, s, r | **0.56-2.5 m/s**<br>w, r, s, h | **semi-supine, bicyle ergometer** | **standing, lateral decubitus, 0.4-1.8 m/s** $^{w}$ | **standing, 0.6 m/s** $^{w}$ | | **sitting** | | **operational data** | **operational data** | **operational data** | **operational data** | **operational data** |
| | **Number of participants** | **n = 6** | | **n = 11** | **n = 12** | | **n = 15** | **n = 14** | | **n = 21** | | **n = 6** | **n = 6** | | **n = 13** | **n = 6** | **n = 6** | **n = 6** | **n = 12** | | **n = 14** | | **n = 2** | | | | |
| | **Control condition** | 1g | μg | 1g | 1g | μg | 1g | 1g | μg | 1g | μg | μg | 1g | μg | 1g | 1g | μg | 1g | 1g | μg | 1g | μg | 1g | 1g | 1g | 1g | 1g |
| **Cardiac** | **Heart rate** [bpm] | | | → | | | ↓* | ↓* | ↑* | ↓* | ↑ LBPP<br>↓ HUT | ↓ 2h, 4h, 5h<br>→ 6h | | | | | ↑ | ↓ | ↓ | ↑ | | | | Ø 90-100;<br>max: 160 | | max: 140-160 | max: 140-160 |
| | **NN interval, meanRR** [ms] | ↓ | ↓ | | | | | | | | | | | | | | | | | | ↑* | ↑ | | | | | |
| | **Heart rate variability (SDNN, stdRR)** [ms] | ↑ | ↓ | | | | | | | | | | | | | | →<br>PNS↓* SNS↑* | | | | ↑ | ↓ | | | | | |
| | **Stroke volume** [ml] | | | → | | | | ↑* | ↓* | ↑* | ↓ | ↓ 2h, 4h, 5h, 6h | | | | | | | | | | | | | | | |
| | **Cardiac output** [l/min] | | | → | | | | | | ↑ | ↓ | ↓ 2h, 4h, 5h, 6h | | | | | | | | | | | | | | | |
| | **Ejection fraction** [%] | | | → | | | | | | | | | | | | | | | | | | | | | | | |
| | **Left ventricular systolic volumes** [ml] | | | | | | | | | | | ↓ 2h<br>↑ 4h, 5h<br>→ 6h | | | | | | | | | | | | | | | |
| | **Left ventricular diastolic volumes** [ml] | | | | | | | | | | | ↓ 2h, 4h, 5h, 6h | | | | | | | | | | | | | | | |
| **Blood Pressure** | **Systolic blood pressure** [mmHg] | ↓* | ↓ | → | | | ↑ | | | ↑ | | ↑ 2h, 4h, 5h, 6h | | | | | ↓* | | | | ↓ | ↓ | | | | | |
| | **Diastolic blood pressure** [mmHg] | ↓* | ↓ | → | | | ↑ | ↑$^{\#}$ | ↑$^{\#}$ | ↓ HUT | | ↑ 2h, 4h, 5h, 6h | | | | | ↓* | | | | ↓ | ↓ | | | | | |
| | **Mean arterial blood pressure** [mmHg] | | | | ↓ foot | ↑ foot | ↑ | | | ↑ | ↑ | ↓ 2h, 4h<br>↑ 5h<br>→ 6h | | | | | ↓* | | → | | ↓ | | | | | | |
| | **Blood pressure ramps** [%] | | | | | | | ↓ | ↓ | | | | | | | | | | | | | | | | | | |
| | **Total peripheral resistance** [mmHg/l/min] | | | | | | | ↑ | ↑* | ↓ | ↑ | ↑ 2h, 4h, 5h, 6h | | | | | | | | | | | | | | | |
| **Haemodynamics** | **Blood flow velocity** [cm/s] | | | | ↑ foot sole | ↓ foot sole | ↑ middle cerebral artery | | | | | | | | | | | | | | | | | | | | |
| | **Venous diameter (jugular)** [cm] | | | ↑ | | | | | | | | | | | | | | | | | | | | | | | |
| | **Venous diameter (femoral)** [cm] | | | ↓ | | | | | | | | | | | | | | | | | | | | | | | |
| | **Venous emptying volume** [ml] | | | | | | | | | | | | ↑ | ↓ | | | | | | | | | | | | | |
| | **Venous emptying time** [s] | | | | | | | | | | | | ↑ | ↓ | | | | | | | | | | | | | |
| | **Bioelectrical thoracic impedance** [Ω] | | | | | | | ↓ | ↑* | ↓ | ↑ LBPP<br>↓ HUT | | | | | | | | | | | | | | | | |
| | **Bioelectrical abdominal impedance** [Ω] | | | | | | | ↓ | ↓* | ↓ LBPP<br>↑ HUT | ↓ | | | | | | | | | | | | | | | | |
| **Respiratory** | **Oxygen consumption** [ml/kg/min] | | | | | | | | | | | | | | | | | ↓ | ↓ | ↑ | | | | | | | |
| | **Carbon dioxide production** [ml/kg/min] | | | | | | | | | | | | | | | | | ↓ rest | | | | | | | | | |
| | **Respiratory rate** [breaths/min] | | | | | | | | | | | | | | | | | ↓ | | | | | | | | | |
| | **Respiratory gas exchange ratio** | | | | | | | | | | | | | | | | | → rest | | | | | | | | | |
| | **Expired minute volume** [l/min] | | | | | | | | | | | | | | | | | ↓ rest | | | | | | | | | |
| **Metabolic** | **Metabolic rate** [kcal/hr], [W/kg] | | | | | | | | | | | | | | | | | ↓* | | | | | Ø 234 | Ø 226-300<br>max: 600 | Ø 250-300<br>command.: 236.6<br>lunar module pilot: 237.5 | Ø 227-303 | Ø 227-303 |
| | **Locomotion efficiency** | | | | | | | | | | | | | | ↓* | ↓ | | | | | | | | | | | |
| | **Cost of transport** [J/kg/min] | | | | | | | | | | | | | | ↓$^{\#}$ | ↓$^{\#}$ | | | | | | | | | | | |
